# Does the punishment fit the crime? Consequences and diagnosis of misspecified detection functions in Bayesian spatial capture–recapture modeling

**DOI:** 10.1002/ece3.8600

**Published:** 2022-02-15

**Authors:** Soumen Dey, Richard Bischof, Pierre P. A. Dupont, Cyril Milleret

**Affiliations:** ^1^ 56625 Faculty of Environmental Sciences and Natural Resource Management Norwegian University of Life Sciences Ås Norway

**Keywords:** Bayesian *p*‐value, detection function, goodness‐of‐fit, Kernel home range area, space use

## Abstract

Spatial capture–recapture (SCR) analysis is now used routinely to inform wildlife management and conservation decisions. It is therefore imperative that we understand the implications of and can diagnose common SCR model misspecifications, as flawed inferences could propagate to policy and interventions. The detection function of an SCR model describes how an individual's detections are distributed in space. Despite the detection function's central role in SCR, little is known about the robustness of SCR‐derived abundance estimates and home range size estimates to misspecifications. Here, we set out to (a) determine whether abundance estimates are robust to a wider range of misspecifications of the detection function than previously explored, (b) quantify the sensitivity of home range size estimates to the choice of detection function, and (c) evaluate commonly used Bayesian *p*‐values for detecting misspecifications thereof. We simulated SCR data using different circular detection functions to emulate a wide range of space use patterns. We then fit Bayesian SCR models with three detection functions (half‐normal, exponential, and half‐normal plateau) to each simulated data set. While abundance estimates were very robust, estimates of home range size were sensitive to misspecifications of the detection function. When misspecified, SCR models with the half‐normal plateau and exponential detection functions produced the most and least reliable home range size, respectively. Misspecifications with the strongest impact on parameter estimates were easily detected by Bayesian *p*‐values. Practitioners using SCR exclusively for density estimation are unlikely to be impacted by misspecifications of the detection function. However, the choice of detection function can have substantial consequences for the reliability of inferences about space use. Although Bayesian *p*‐values can aid the diagnosis of detection function misspecification under certain conditions, we urge the development of additional custom goodness‐of‐fit diagnostics for Bayesian SCR models to identify a wider range of model misspecifications.

## Graphical abstract

We investigated the effect of misspecifiying the detection function in spatial capture–recapture models and discovered that such misspecification can cause erroneous home range size inferences, whereas population size estimates are mostly unaffected. In addition, we explored and showcased the utility and limitations of various Bayesian *p*‐values for diagnosing misspecifications of the detection function.

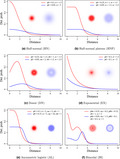

## 1 INTRODUCTION

Spatial capture–recapture (SCR) models are now routinely used to monitor wildlife populations, estimate parameters of applied importance such as population size and space use (Borchers & Efford, 2008; Royle et al., 2014, 2018), and inform their management and conservation (Bischof, Milleret, et al., 2020; López‐Bao et al., 2018). These are hierarchical models that use the spatial information from repeated individual encounters to estimate density and space use parameters in wildlife populations, while accounting for imperfect detection. The Bayesian paradigm and accessible programming languages (de Valpine et al., 2017; Plummer, 2003) provide a convenient framework for developing and fitting custom SCR models (Bischof, Milleret, et al., 2020; Turek et al., 2021), and we are experiencing a surge of innovation in Bayesian SCR models of growing scope and complexity to study landscape connectivity, movement, and other space use dynamics (Royle et al., 2018).

Spatial capture–recapture can provide managers and policymakers with multiple important parameters, not only abundance (Royle et al., 2018). It is imperative to minimize the risk of erroneous inferences finding their way into the resource management decision‐making process. Specifically, there is a dearth of studies that systematically evaluate the consequences of model misspecifications on space use parameters (e.g., home range size) and reliability of commonly used diagnosis tools in Bayesian SCR models (Royle et al., 2009, 2014; Russell et al., 2012).

In this study, we focus on the consequences and diagnosis of misspecifications of a core component of SCR models: the detection function. Detection probability in SCR is modeled as a function of the distance between latent individual activity centers (ACs) and detection locations. Often, detection patterns in SCR studies emerge from the movement of individuals about their home ranges. In these cases, the shape of the detection function can be interpreted as a reflection of an individual's space use around its activity center: an individual is more likely to be detected in areas in which it spends more time.

The half‐normal detection function (HN) is the most common detection function in SCR and assumes a bivariate normal model of animal space use with a monotonic decay in detection probability as distance from the AC increases (Efford, 2004). However, animal space use and home range configurations can vary substantially between (Ofstad et al., 2016) and even within species (Efford & Mowat, 2014) and it is unlikely that “one size fits all,” as far as SCR detection functions are concerned. For example, territorial species may spend a disproportionate amount of time patrolling and scent marking their territorial boundaries (Langergraber et al., 2017), leading to a bimodal or donut‐shaped space use profile. Conversely, species or demographic groups with long exploratory forays (Zeale et al., 2012) may exhibit long‐tailed space use distributions. Species with relatively even space use throughout a clearly defined home range (Pearce et al., 2013) may represent another deviation from the half‐normal model, exhibiting a plateau in the utilization distribution followed by a rapid decline in utilization near the edge of the home range.

Although there are indications that density estimates produced by SCR models are robust to misspecifications of the detection function (Efford, 2004; Russell et al., 2012), this has only been explored for a limited range of scenarios. Furthermore, detection function misspecifications may have consequences for inferences about space use and movement, which feature increasingly in the SCR literature (Bischof et al., 2017; Royle, Chandler, Gazenski, et al., 2013). Even in the absence of systematic bias, misspecifications could affect the associated precision and coverage probability (Bischof, Dupont, et al., 2020).

While simulations can inform about the potential consequences of model misspecifications, they cannot be used to identify them in empirical situations. Goodness‐of‐fit (GOF) testing offers a formal tool for diagnosing violations of assumptions and is an important part of statistical analysis as it reduces the risk of drawing erroneous inference (Pradel et al., 2005). Bayesian *p*‐values are frequently used to assess GOF in Bayesian modeling and do so by measuring the systematic dissimilarity between observed data and model‐predicted data (i.e., replicated data Gelman et al., 2014). Although Bayesian *p*‐values have been used previously for assessing GOF of different SCR model components (Ergon & Gardner, 2014; Russell et al., 2012), their efficacy in diagnosing misspecified detection functions in SCR models has yet to be explored.

Using simulations, we evaluated the consequences of detection function misspecifications on key SCR parameter estimates and assessed whether Bayesian *p*‐values can be used to diagnose such misspecifications. First, we quantified the impact of the choice of detection function—relative to the data‐generating function—on SCR‐derived estimates of home range size and population size. Then, we calculated and compared a suit of Bayesian *p*‐values and assessed their ability to reveal misspecifications. We discuss the implications of our results in the context of the choices and challenges faced by practitioners using SCR.

## 2 METHODS

### 2.1 General approach

**FIGURE 1:**
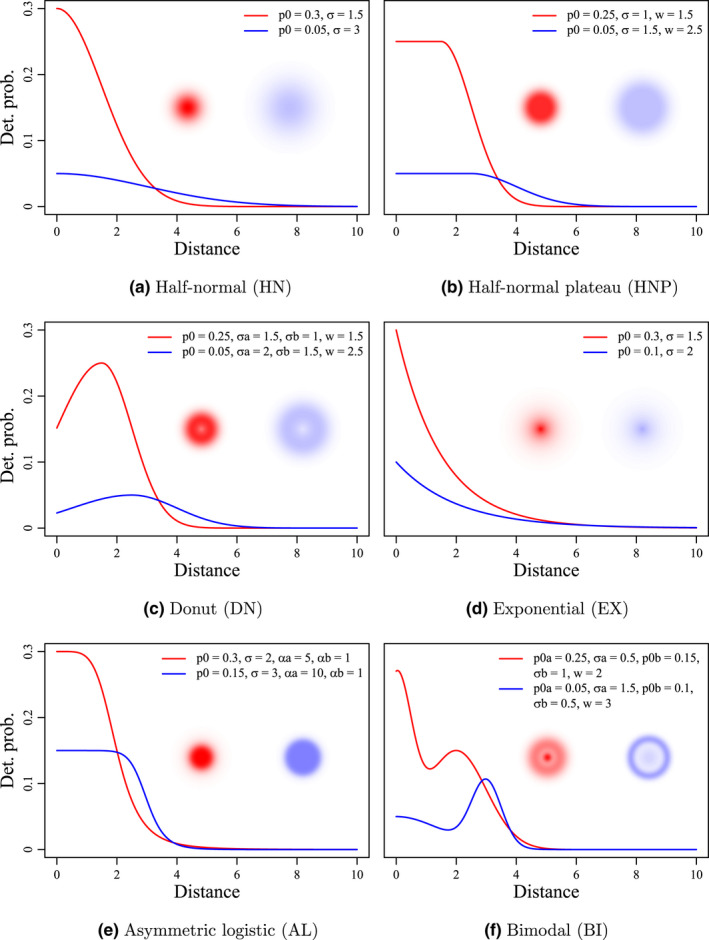
Visualization of six different detection functions (detection probability as a function of distance between the detector and individual activity center), both as kernel density profiles and raster maps. Realization of two different parameter sets is shown for each detection function, with red lines and shading correspond to parameter set 1, whereas blue lines and shading correspond to parameter set 2. Parameter values used for each detection function (see main text for descriptions) are provided in the legend of each plot. Distances are provided in arbitrary distance units (du)

We conducted a simulation study to assess the consequences of misspecifying the detection function in SCR models. Six different detection functions were used to simulate contrasting animal space use patterns and generate corresponding spatial capture–recapture data (Figure 1). We then fitted three SCR models differing in their detection function (half‐normal, exponential, or half‐normal plateau; see Section 2.3.2 below) to the simulated data sets. We assessed the robustness of the models using relative bias, coefficient of variation, and coverage probability of the 95% credible intervals of population size and home range size estimates. Finally, we calculated a suite of Bayesian *p*‐values and compared their ability to identify misspecifications of the detection function.

### 2.2 SCR model description

A single‐season SCR model typically consists of a submodel describing the spatial distribution of individual ACs in a given area (habitat) and an observation submodel describing individual detection probability in space, conditional on AC locations. Consider *N* individuals that reside in a bounded habitat $V\subset R^{2}$ where each individual is assumed to move randomly around its AC, viz., s∈V. Following a homogeneous point process, each individual AC is assumed to be uniformly distributed across the habitat V (Royle et al., 2014).

We used a data augmentation approach to model the number of individuals *N* in the habitat V (Royle et al., 2009). We chose a large integer *M* to bound *N* and introduced a vector of *M* latent binary variables *z* = (*z*_1_, *z*_2_, …, *z_M_*) such that *z_i_* = 1 if individual *i* is a member of the population and *z_i_* = 0 otherwise. We assume that each *z_i_* is a realization of a Bernoulli trial with parameter *ψ*, the inclusion probability.

We considered *J* detectors located within V, active during a single sampling occasion. Recorded detections (*y_ij_* 's) are binary, that is, *y_ij_* = 1 if the *i*‐th individual is detected at the *j*‐th detector and *y_ij_* = 0 otherwise. Note that an individual can be detected at multiple detectors within the same sampling occasion. If *n* individuals are detected during the capture–recapture survey, **Y**_obs_ is of dimension *n* × *J*. As part of the data augmentation approach, the SCR data set **Y**_obs_ = ((*y_ij_*)) is supplemented with a large number of “all‐zero” detection histories. The zero‐augmented data set **Y** (dimension *M* × *J*) is constructed as follows:

(1) $$Y=\left(\begin{array}{c} Y_{\text{obs}}\\ Y_{\text{rem}} \end{array}\right),$$

where **Y**_rem_ denotes the array of “all‐zero” detection histories with dimensions (*M* − *n*) × *J*.

A Bernoulli model, conditional on *z_i_*, is assumed for each observation *y_ij_*:

(2) $$y_{\mathrm{ij}}∼\text{Bernoulli}\left(p_{\mathrm{ij}}z_{i}\right),$$

where *p_ij_* denotes the detection probability of the *i*‐th individual at the *j*‐th detector. The detection probability *p_ij_* is modeled as a function of the Euclidean distance *d_ij_* between the detector location *x_j_* and individual AC location *s_i_*

(3) $$p_{\mathrm{ij}}=p\left(d_{\mathrm{ij}}\right),$$

where

(4) $$d_{\mathrm{ij}}=d\left(s_{i},x_{j}\right)=∥s_{i}‐x_{j}∥.$$

This generic formulation of *p_ij_*, given in (3), accounts for individual‐ and detector‐level heterogeneity in detection probability due to their relative position in space. In addition, this implementation allows the user to specify the shape of the detection function *p* in terms of *d_ij_*. The detection function is the focus of this manuscript and is discussed in more detail in Section 2.3.2.

### 2.3 Simulation design

#### 2.3.1 Habitat and detectors

**FIGURE 2:**
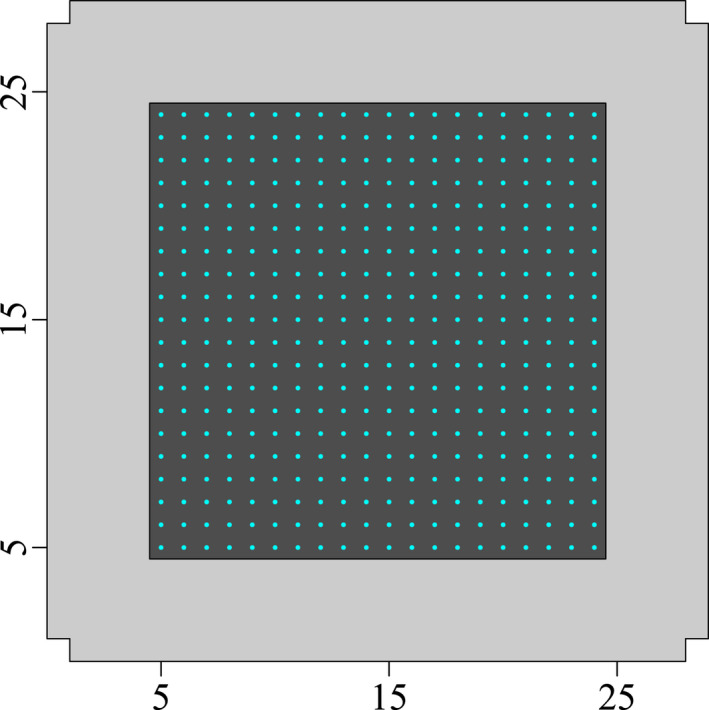
Illustration of the habitat and the detector grid configuration used in spatial capture simulations: The detector array is a rectangular grid of 20 × 20 detectors (blue dots, 400 detectors total). The habitat covers an area of 29 × 29 distance units, including the detector region (dark grey area) and a 5‐distance units unsampled buffer (light grey area) surrounding the detector array

We simulated a 20 × 20 detector array (*J* = 400 detectors) with 1 distance unit (du) spacing between neighboring detectors centered on a 29 × 29 du habitat V. This configuration results in a 5‐du habitat buffer around the outermost detectors (Figure 2). Although the number of detectors used here is larger than what is achieved in some study systems (e.g., most camera trapping studies), it was selected to ensure good spatial coverage and a sufficient number of recaptures to allow us to reliably quantify the effect of detection function misspecification.

#### 2.3.2 Detection functions

We considered six functions with substantial variation in their shapes (Table 1 and Figure 1). In SCR modeling, it is a common practice to assume that individual space use is circular, highest at the AC location and gradually decays with distance from AC (Royle et al., 2014). Both the half‐normal (HN) and exponential (EX) detection functions (Table 1, Figure 1a,d) reflect this behavior and are frequently used in SCR studies, with the half‐normal being the most popular choice. In both functions, *p*_0_ denotes the baseline detection probability (i.e., the detection probability at the exact location of the AC) and the scale parameter *σ* quantifies the spatial extent of animal space use around its AC.

**TABLE 1:** Parameter values of the six detection functions used for simulating spatial capture–recapture data. Also shown are the corresponding 95% quantile home range area and number of detected individuals (mean, 2.5% and 97.5% quantiles) for two parameter sets

Detection function	Equation	Parameters	Parameter set 1			Parameter set 2		
			Parameter values	95% HR area (du sq.)	No. of detected individuals	Parameter values	95% HR area (du sq.)	No. of detected individuals
Half‐normal (HN)	$p_{\text{HN}\;}\left(d\right)=p_{0}\exp\left(‐\frac{d^{2}}{2\sigma^{2}}\right)$	*p*_0_ ∈ (0, 1), *σ* > 0 (Figure 1a)	*p*_0_ = .3, *σ* = 1.5	42.35	123 (111, 137)	*p*_0_ = .05, *σ* = 3	169.40	123 (110, 137)
Exponential (EX)	$p_{\text{EX}\;}\left(d\right)=p_{0}\exp\left(‐\frac{d}{\sigma }\right)$	*p*_0_ ∈ (0, 1), *σ* > 0 (Figure 1d)	*p*_0_ = .3, *σ* = 1.5	158.87	136 (123, 149)	*p*_0_ = .1, *σ* = 2	274.48	117 (104, 132)
Half‐normal plateau (HNP)	$\begin{array}{cc} p_{\text{HNP}\;}\left(d\right) & =p_{0},d<w\;\text{and}\\ & =p_{0}\exp\left(‐\frac{{\left(d‐w\right)}^{2}}{2\sigma^{2}}\right),d\geq w \end{array}$	*p*_0_ ∈ (0, 1), *σ* > 0, *w* ≥ 0 (Figure 1b)	*p*_0_ = .25, *σ* = 1, *w* = 1.5	39.50	133 (123, 146)	*p*_0_ = .05, *σ* = 1.5, *w* = 2.5	95.75	126 (114, 138)
Asymmetric logistic (AL)	$p_{\text{AL}\;}\left(d\right)=p_{0}{\left[1+{\left(d/\sigma \right)}^{\alpha_{a}}g\left(d\right)+{\left(d/\sigma \right)}^{\alpha_{a}\alpha_{b}}\left(1‐g\left(d\right)\right)\right]}^{‐1},$ where $g\left(d\right)={\left\{1+{\left(d/\sigma \right)}^{\nu }\right\}}^{‐1},$ $\nu =\frac{2\left|\alpha_{a}\right|\alpha_{b}}{1+\alpha_{b}}$	*p*_0_ ∈ (0, 1), *σ* > 0, $\alpha_{a}\in R$, *α_b_* > 0 (Figure 1e)	*p*_0_ = .3, *σ* = 2 *α_a_* = 5, *α_b_* = 1	57.82	127 (113, 141)	*p*_0_ = .15, *σ* = 3 *α_a_* = 10, *α_b_* = 1	40.92	127 (115, 139)
Donut (DN)	$p_{\text{DN}}\left(d\right)=p_{0}\exp\left(‐\frac{{\left(d‐w\right)}^{2}}{2\sigma_{a}^{2}}\right),d<w,$ $p_{\text{DN}}\left(d\right)=p_{0}\exp\left(‐\frac{{\left(d‐w\right)}^{2}}{2\sigma_{b}^{2}}\right),d\geq w$	*p*_0_ ∈ (0, 1), *σ_a_* > 0, *σ_b_* > 0, *w* ≥ 0 (Figure 1c)	*p*_0_ = .25, *σ_a_* = 1.5 *σ_b_* = 1, *w* = 1.5	39.98	133 (120, 146)	*p*_0_ = .05, *σ_a_* = 2 *σ_b_* = 1.5, *w* = 2.5	97.14	125 (115, 137)
Bimodal (BI)	$\begin{array}{cc} p_{\text{BI}\;}\left(d\right) & =p_{0a}\exp\left(‐\frac{d^{2}}{2\sigma_{a}^{2}}\right)\\ & \;+p_{0b}\exp\left(‐\frac{{\left(d‐w\right)}^{2}}{2\sigma_{b}^{2}}\right) \end{array}$	*p*_0_ *_a_* ∈ (0, 1), *σ_a_* > 0, *p*_0_ *_b_* ∈ (0, 1), *σ_b_* > 0, *w* ≥ 0 (Figure 1f)	*p*_0_ *_a_* = .25, *σ_a_* = 0.5 *p*_0_ *_b_* = .15, *σ_b_* = 1, *w* = 2	49.64	133 (120, 147)	*p*_0_ *_a_* = .05, *σ_a_* = 1.5 *p*_0_ *_b_* = .1, *σ_b_* = 0.5, *w* = 3	46.81	121 (107, 136)

To allow greater flexibility in individual space use, we considered two additional detection functions. The half‐normal plateau (HNP) is an extension of the half‐normal detection function with an additional non‐negative parameter *w* for the radius of uniform activity (Table 1, Figure 1b). The asymmetric logistic detection function (AL) is an extension of the logistic curve model with an additional parameter *α_b_* for a second curvature (Table 1, Figure 1e, also see Ricketts & Head, 1999). The other parameters of the AL function are *p*_0_, the baseline detection probability, *α_a_*, the first curvature parameter, and *σ*, the distance from the AC where the asymmetric logistic detection function takes the value *p*_0_/2.

Finally, we included two detection functions which allowed an increase in space use with distance from AC (e.g., common pipistrelle bats: *Pipistrellus pipistrellus;* Nicholls & Racey, 2006). The donut function (DN) is characterized by a single peak with highest detection probability *p*_0_ at a distance *w* from the AC (Table 1, Figure 1c). The two tails on either sides of the peak correspond to two half‐normal density curves with mean *w* and scale parameters *σ_a_* and *σ_b_*. The bimodal function (BI) is a stochastic mixture of two univariate normal densities with means 0 and *w*(>0) and scale parameters *σ_a_* and *σ_b_*, with mixture weights *p*_0_ *_a_* and *p*_0_ *_b_*, respectively (Table 1, Figure 1f).

### 2.4 Scenarios

We simulated single‐season spatial capture–recapture data sets (Section 2.2) for *N* = 200 individuals with the six detection functions and two sets of detection function parameters (Table 1, Figure 1), leading to 12 different simulation scenarios. The two parameter sets were chosen to result in a comparable number of detected individuals (around 60%–70%) but different home range sizes (corresponding to the distance that delimits 95% of the area under the detection kernel) and different total detection counts (i.e., $\sum_{i=1}^{M}\sum_{j=1}^{J}y_{\mathrm{ij}}$; Appendix S2: Figure S1). Like the number of detectors, the detection parameters were chosen to result in a sufficiently high number of recaptures in the simulated data, thereby boosting our ability to detect potential patterns arising from detection function misspecification.

Estimated home range sizes differed between the three fitted models because of the different shapes of the detection functions (Table 1). We chose parameter sets that resulted in simulated SCR data with similar proportions (around 60%–70%) of individuals detected (Table 1) in order to avoid confounding between detection functions in information contents.

Due to computation limitations, we did not fit SCR models with all six detection functions used during simulation (see Section 2.3.2). Instead, we fitted the two most common ones: half‐normal (HN) and exponential (EX), as well as the half‐normal plateau (HNP) for its expected flexibility. For each of the three models to be fitted and for each of the 12 scenarios, we simulated 50 independent SCR data sets, resulting in 1800 simulated data sets.

To ensure realistic simulations, we computed a population‐level index of home range overlap *S* = *DH*, where *D* denotes the population density and *H* denotes the home range size (Damuth, 1981, based on the 95% density of space use distribution). Home range overlap *S* (average number of individuals using a single home range area) ranged from 9 to 65 in our simulation study (with *D* = 0.24 animals per du sq).

### 2.5 Model fitting

We fitted models using Markov chain Monte Carlo (MCMC) simulations with NIMBLE (de Valpine et al., 2017) in R (R Core Team, 2019). To reduce computation time, we implemented the local evaluation approach (Milleret et al., 2019; Turek et al., 2021). We ran three chains of 15,000 iterations including an initial burn‐in phase of 5000 iterations. MCMC convergence of each model was monitored using the Gelman‐Rubin convergence diagnostics $\hat{R}$ (Gelman et al., 2014).

During preliminary analyses, we observed slow mixing of the Markov chains for parameters *σ* and *w* of the HNP detection function with the standard MCMC within Gibbs sampler. To improve mixing, we used the recycling Gibbs sampler (Martino et al., 2018) for these parameters. R code for implementing simulations and fitting the single‐season SCR model with different detection functions is provided in Data S1 and S2.

### 2.6 Consequences of misspecification

#### 2.6.1 Deriving home range size

The parameters of the different detection functions used in this study cannot be directly compared between functions. We therefore based our comparison of the different models on the estimates of home range size that can be derived from each detection function estimate. This is also the parameter of interest to practitioners. All detection functions *p*(*x*, *s*) used during fitting (Table 1) are proportional to the probability density function of a bivariate distribution *g*(*x*|*s*) that represents individual space use distribution:

(5) $$g\left(x|s\right)=\frac{p\left(x,s\right)}{\sum_{x\in V}p\left(x,s\right)}.$$

For any such space use probability distribution, we can find the quantile *r_α_* such that *α*% of all movements lie within the circle of radius *r_α_* centered on *s*. We can then define the *α*% home range area as *A_α_*, the set of all points x∈V such that ||*x* − *s*|| ≤ *r_α_*. Assuming a circular home range, the size of *A_α_* is then simply calculated as $\pi r_{\alpha }^{2}$. The *α*% home range size can therefore be derived by finding *r_α_* such that $\sum_{x\in A_{\alpha }}g(x|s)\leq \alpha$. Here, we used a bisection algorithm to find the root of the above optimization problem (i.e., to find *r_α_*; see Data S1 and S2 for the R code to derive home range size; Corliss, 1977).

For the half‐normal detection function *p*_HN_(*x*, *s*) = *p*_0_ exp(−0.5*σ* ^−2^ ||*x* − *s*||^2^), an analytical solution exists to calculate *r_α_*. Since *σ* ^−2^ ||*x* − *s*||^2^ follows a chi‐square distribution with 2 degrees of freedom, *r_α_* can be calculated as $\sigma \sqrt{q\left(\alpha ,2\right)}$ where *q*(*α*, 2) is the *α*% quantile of a chi‐square distribution with 2 degrees of freedom (Royle et al., 2014). As the analytical solution is more accurate and faster to obtain than numerical approximations, we analytically derived home range size for the HN model but used numerical approximations for the other five detection functions for which no simple analytical solution exists. In order to compare the different models, we calculated home range sizes using a thinned sample (thinning rate = 10) combining all the MCMC chains, thus producing posterior distributions of the *α*% home range size (e.g., *α* = 95 or 50).

#### 2.6.2 Deriving population size *N*

Population size is a derived parameter which follows a binomial distribution with parameters *M* and ψ, $N=\sum_{i=1}^{M}z_{i}$. The parameter *ψ* gives the probability that an arbitrary individual from the set of *M* individuals is a member of the population. For model fitting, we set *M* to 400 for all scenarios.

#### 2.6.3 Model performance measures

We used relative bias, coefficient of variation, and coverage probability to evaluate the effect of detection function misspecifications on population size and home range size estimators. Suppose {*θ* ^(^ *^r^* ^)^: *r* = 1, 2, …, *R*} denotes a set of MCMC draws from the posterior distribution of a scalar parameter *θ*.

##### Relative bias

Relative bias (RB) is calculated as

(6) $$\hat{\text{RB}}\left(\theta \right)=\frac{\hat{\theta }‐\theta_{0}}{\theta_{0}},$$

where $\hat{\theta }$ denotes the posterior mean $\frac{1}{R}\sum_{r=1}^{R}\theta^{\left(r\right)}$ and *θ_0_* denotes the true (simulated) value.

##### Coefficient of variation

Precision was measured by the coefficient of variation (CV):

(7) $$\hat{\text{CV}}\left(\theta \right)=\frac{\hat{\text{SD}}\left(\theta \right)}{\hat{\theta }},$$

where $\hat{\text{SD}}\left(\theta \right)=\sqrt{\frac{1}{R}\sum_{r=1}^{R}{\left(\theta^{\left(r\right)}‐\hat{\theta }\right)}^{2}}$ is the posterior standard deviation of parameter *θ*.

##### Coverage probability

Coverage probability was computed as the proportion of model fits (to the 50 simulations within a set, see Section 2.4) for which the estimated 95% credible interval of the estimate (CI) contained the true value of *θ*.

### 2.7 Goodness‐of‐fit testing

We assessed the goodness‐of‐fit of SCR models using Bayesian *p*‐values (Gelman et al., 2014). It is a model checking procedure which measures the dissimilarity between the observed data **Y** and the model‐predicted data **Y** ^rep^. The computation of Bayesian *p*‐values requires specifying a discrepancy measure *T* chosen to reflect aspects of the model that are to be checked.

In practice, posterior replicates of a SCR data set **Y** are obtained by drawing one replicated data set $Y_{r}^{\text{rep}}$ from the fitted model for each posterior simulation of parameter vector **θ** *_r_* (*r* = 1, 2, …, *R*), where *R* denotes the number of posterior MCMC samples. Consequently, two sets of discrepancy measures (*T*(**Y**, **θ**_1_), *T*(**Y**, **θ**_2_), …, *T*(**Y**, **θ** *_R_*))′ and $(T\left(Y_{1}^{\text{rep}},\theta_{1}\right),T{\left(Y_{2}^{\text{rep}},\theta_{2},\ldots ,T\left(Y_{R}^{\text{rep}},\theta_{R}\right)\right)}^{\prime }$ are generated. The Bayesian *p*‐value is calculated as the proportion of times the replicated discrepancy measure $T(Y_{r}^{\text{rep}},\theta_{r})$ is greater than the observed quantity *T*(**Y**, **θ** *_r_*):

(8) $$\text{Bayesian}\;p\text{-value}\approx \frac{1}{R}\sum_{r=1}^{R}I\left\{T\left(Y_{r}^{\text{rep}},\theta_{r}\right)>T\left(Y,\theta_{r}\right)\right\},$$

where *I*(*x*) is an indicator function taking the value 1 if *x* is true and 0 otherwise.

If the model fit is adequate, the Bayesian *p*‐value should be near .5 as the discrepancy measure in the replicated data set would be equally likely greater or less than the observed measure, thus indicating that the model‐predicted data are consistent with the observed data with respect to the aspect the discrepancy measure is designed to check (but the converse is not always true, see Section 4.3). The general recommendation to identify a lack of fit with Bayesian *p*‐values is to check whether or not they fall outside the interval (.1, .9), also in SCR (Royle et al., 2014).

Although a range of different discrepancy measures exist, currently, no universal discrepancy measure is available for SCR models. We therefore implemented four different discrepancy measures to compare their ability to identify misspecifications in the detection function. For notational convenience, we suppressed the dependency of the discrepancy measure on the data set and the parameter henceforth. We used the Freeman–Tukey (FT) measure for its applicability for sparse data sets due to the variance stabilizing square root transformation (Brooks et al., 2000):

(9) $$T_{\text{FT}}=\sum_{i=1}^{M}\sum_{j=1}^{J}{\left(\sqrt{y_{\mathrm{ij}}}‐\sqrt{E_{\mathrm{ij}}}\right)}^{2},$$

where *y_ij_* and *E_ij_* denote the capture–recapture observation for individual *i* at the *j*‐th detector and its expected value under the fitted model, respectively. We also used two versions of this statistic pooled at the individual or detector level:

(10) $$T_{\text{FT-I}}=\sum_{i=1}^{M}{\left(\sqrt{y_{i0}}‐\sqrt{E_{i0}}\right)}^{2},$$

and

(11) $$T_{\text{FT-D}}=\sum_{j=1}^{J}{\left(\sqrt{y_{0j}}‐\sqrt{E_{0j}}\right)}^{2},$$

where *y_i_*_0_ = ∑*_j_y_ij_* denotes the number of observations for individual *i* across the *J* detectors and *y*_0_ *_j_* = ∑*_i_y_ij_* denotes the number of individuals observed at detector *j* with *E_i_*_0_ = ∑*_j_E_ij_* and *E*_0_ *_j_* = ∑*_i_E_ij_*.

In addition, we used Pearson's *chi‐square* metric (Gelman et al., 2014):

(12) $$T_{P}=\sum_{i=1}^{M}\sum_{j=1}^{J}\frac{{\left(y_{\mathrm{ij}}‐E_{\mathrm{ij}}\right)}^{2}}{E_{\mathrm{ij}}}.$$

We calculated all Bayesian *p*‐values using a thinned sample (thinning rate = 10) of all MCMC samples drawn during fitting of a given model.

## 3 RESULTS

**FIGURE 3:**
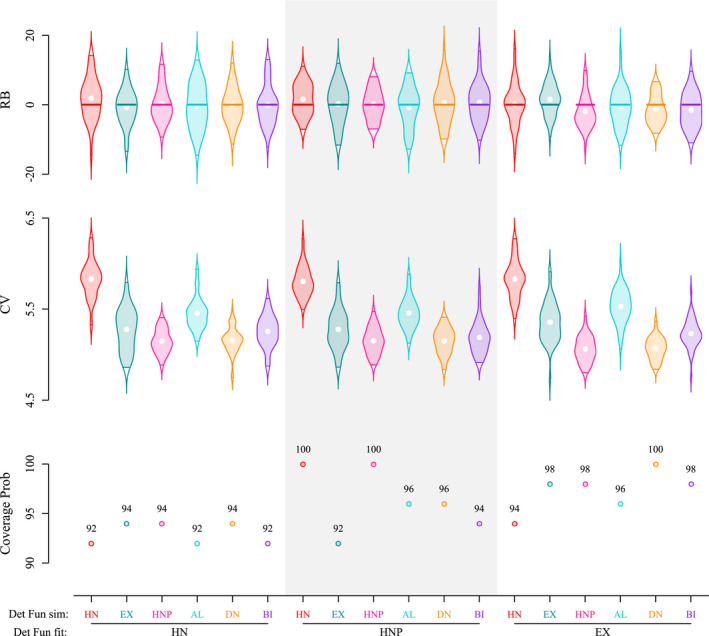
Posterior summaries of population size *N* derived using spatial capture–recapture in parameter set 1. Results compare relative bias (RB, in %), coefficient of variation (CV, in %), and 95% coverage probability (in %) for different pairings of simulated and fitted detection functions. Detection functions include the half‐normal (HN), exponential (EX), half‐normal plateau (HNP), asymmetric logistic (AL), donut (DN), bimodal (BI). Violins represent the distribution of RB/CV from 50 simulations

All MCMC samples of the parameters of interest (e.g., *N*, *α*% home range size) were obtained after ensuring proper mixing and convergence, with $\hat{R}$ values below 1.1. When correctly specified, that is, fitted with the detection function used for simulation (in our case, with HN. HNP or EX functions), both population size and home range size were estimated without significant bias (average RB between 0% and 4%), with good precision (CV < 10%) and nominal coverage (approx. .95; Figure 3).

### 3.1 Consequences of misspecification

#### 3.1.1 Home range area

**FIGURE 4:**
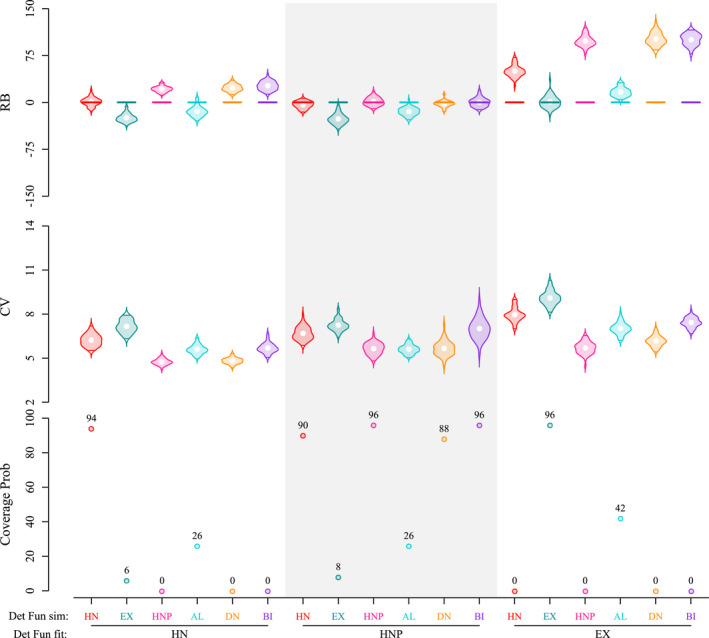
Posterior summaries of home range area derived using spatial capture–recapture in parameter set 1. Home range area was estimated as the 95% kernel of the utilization distribution from the realization of detection function used during model fitting. Results compare relative bias (RB, in %), coefficient of variation (CV, in %), and 95% coverage probability (in %) for different pairings of simulated and fitted detection functions. Detection functions include the half‐normal (HN), exponential (EX), half‐normal plateau (HNP), asymmetric logistic (AL), donut (DN), and bimodal (BI). Violins represent the distribution of RB/CV from 50 simulations

**FIGURE 5:**
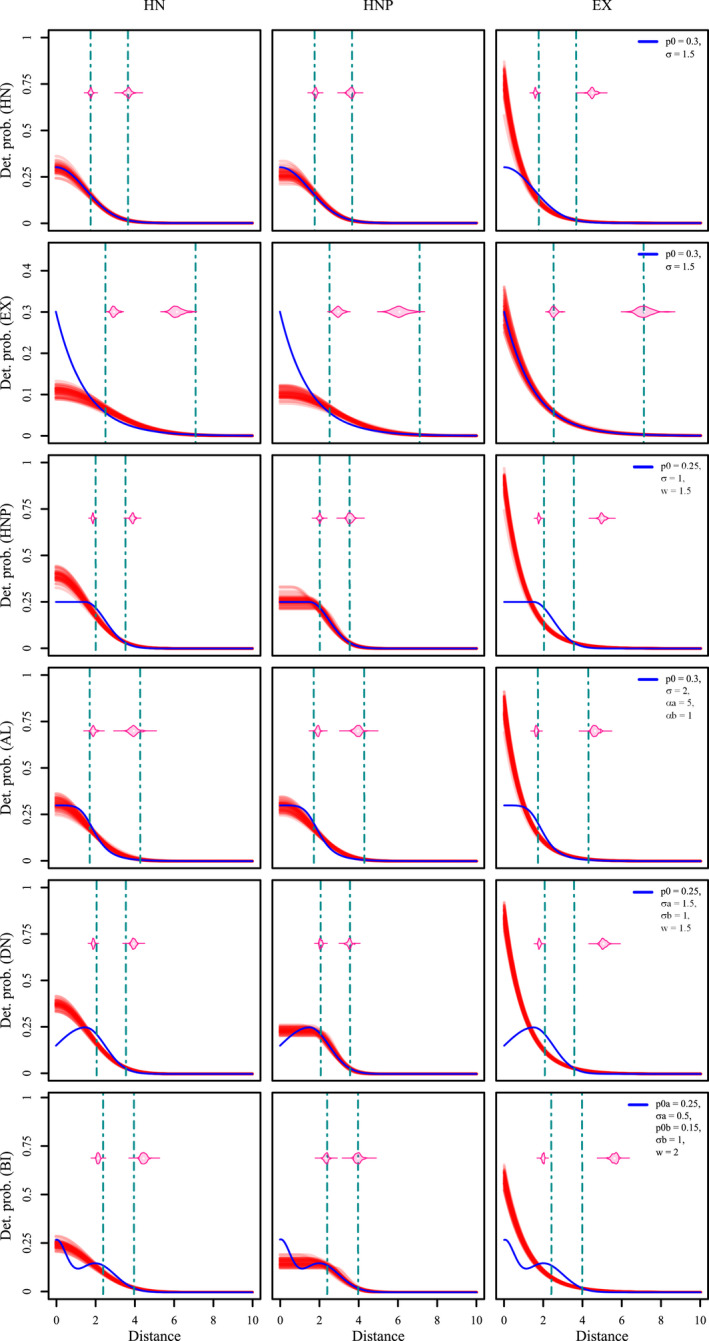
Comparison of estimated detection functions (“red” lines) and the estimates of home range radius (50% and 95% quantiles, “pink” violins representing the distribution from 50 simulations) with the “true” detection function (“blue” line) and “true” home range radius (“cyan” vertical dashed lines) for different scenarios in parameter set 1. Rows correspond to the “true” detection function used to simulate the SCR data sets, and columns represent the detection function used to fit the SCR model. Parameter estimates of each model fitting were used to estimate the fitted detection function and they are plotted as a function of distance in arbitrary distance units

Most misspecifications of the detection function led to erroneous 95% kernel home range size estimates (Figures 4 and 5, Appendix S2: Figures S2 and S5). Although we did not notice any specific pattern in the coefficients of variation, home range size estimates were biased and had very low coverage for both parameter sets with the misspecified HN and EX models.

The EX model overestimated home range size by up to 170% (mean RB between 50% and 170%) and coverage probabilities decreased to 0 when fitted to the HN, HNP, DN, or BI data. The only exception was the AL data for parameter set 1, where relative bias was lower (mean RB = 17%) and coverage higher (42%).

The HN model also overestimated home range size, but to a lesser extent (mean RB between 21% and 71%), when fitted to the HNP, DN, or BI data, with coverage probabilities between 0% and 32%. The HN model underestimated home range size when fitted to the EX data (mean RB of −25% and −22% for parameter sets 1 and 2), with coverage probabilities of 6% and 38% respectively. The pattern was also different for the AL data, with negative bias (mean RB = −14%; coverage = 26%) with parameter set 1 and positive bias with parameter set 2 (mean RB = 31%; coverage = 0%).

The HNP model was most forgiving and accommodated HN, DN, and BI data (mean RB between −7% and 1%, coverage ≥ 88%). However, home range size was underestimated (mean RB = −26% and −31% for parameter sets 1 and 2, respectively) with low coverage probability (≤ 10%) when fitted to the EX data. Again, the pattern differed for the AL data with a negative bias (mean RB = −15%; coverage = 26%) with parameter set 1 and a small positive bias with parameter set 2 (mean RB = 7%; coverage = 78%).

Conversely, when true space use pattern followed an EX or HNP model, we observed the largest average bias in home range size estimation when the detection function in the fitted model was misspecified (mean RB < −22% for EX and > 21% for HNP, see Table S3 in Appendix S1). When true space use patterns followed DN or BI models, we observed moderate bias in home range size while fitting with HN or EX models (mean RB > 23% for DN and > 26% for BI), but home range size was estimated with negligible bias when fitting with HNP model (absolute mean RB < 2%).

#### 3.1.2 Population size

We detected no pronounced effect of the choice of detection function (HN, HNP, EX) on relative bias, precision, or coverage of *N* estimates, regardless of the detection function used for simulation (Figure 3 and Appendix S2: Figure S3). Average RB of *N* ranged between −2% and 2% for all three models with parameter set 1 and between −2% and 3% for parameter set 2. Average CV were also comparable for all models (avg. CV = 5.3% and 6.5% for parameter set 1 and 2 respectively), and coverage exceeded 93% for all scenarios.

### 3.2 Goodness‐of‐fit

**FIGURE 6:**
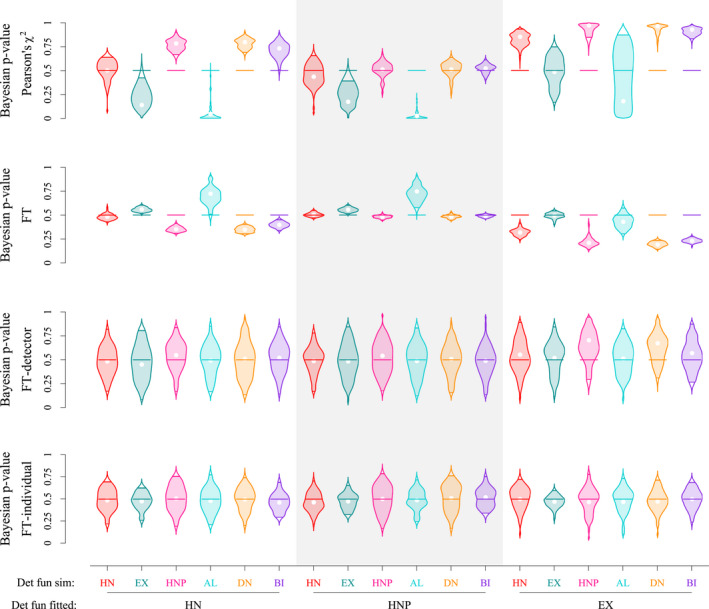
Estimates of Bayesian *p*‐values from different metrics: Freeman–Tukey (FT), Pearson's *chi‐squared*, FT metric based on individual level count (FT‐I), and FT metric based on detection level count (FT‐D). Graphs compare the Bayesian *p*‐value estimates between different pairings of simulated and fitted detection functions for parameter set 1. Each violin represents the distribution of Bayesian *p*‐values for a specified metric from 50 simulations

Bayesian *p*‐values for individual or detector‐level counts (*T*_FT‐I_ and *T*_FT‐D_) were centered on .5 and highly dispersed, thus failing to reveal misspecifications. Bayesian *p*‐values based on individual‐ and detector‐specific detections were less centered on .5 and less dispersed, thus more useful to detect potential misspecifications (Figure 6 and Appendix S2: Figure S4). We also found that Bayesian *p*‐values *p*(FT) showed higher concentration around .5 compared with *p*(*χ* ^2^).

Both *p*‐values showed no evidence of lack of fit when the fitted model matched the simulation. Taking both parameter sets into account, average *p*(*χ* ^2^) were .49 (HN model, CV 19.9%), .51 (HNP model, CV 12.2%), .50 (EX model, CV 26%); and average *p*(FT) were .49 (HN model, CV 5.1%), .49 (HNP model, CV 3.4%), .50 (EX model, CV 4.4%).

The most pronounced lack of fit was observed for the EX model fitted to the HN, HNP, DN, or BI data (mean *p*(*χ* ^2^) between .84 and .96, mean *p*(FT) between .19 and .32 for parameter set 1, mean *p*(*χ* ^2^) between .7 and .82, mean *p*(FT) between .26 and .48 for parameter set 2). For the AL data, the pattern differed between parameter set 1 (mean *p*(*χ* ^2^) = .32, mean *p*(FT) = .43) and parameter set 2 (mean *p*(*χ* ^2^) = .89, mean *p*(FT) = .48).

Mean *p*(*χ* ^2^) were > .5 for the HN model fitted to the HNP, DN, and BI data (mean *p*(*χ* ^2^) between .72 and .79 for parameter set 1 and between .59 and .69 for parameter set 2). As mentioned above, the pattern was inverted for *p*(FT) (mean *p*(FT) between .31 and .34 for parameter set 1 and between .43 and .47 for parameter set 2). Mean *p*(*χ* ^2^) were < .5, and *p*(FT) > .5 when fitted to the EX data (mean *p*(*χ* ^2^) = .18 and .35, and mean *p*(FT) = .56 and .52 for parameter sets 1 and 2, respectively). When fitted to the AL data, the pattern was again inverted between parameter sets 1 and 2, showing evidence of a lack of fit for parameter set 1 only (mean *p*(*χ* ^2^) = .04 and .67, and mean *p*(FT) = .71 and .41 for parameter sets 1 and 2, respectively).

Bayesian *p*‐values clustered around .5 for the HNP model fitted to the HN, DN, and BI data (mean *p*(*χ* ^2^) between .44 and .52, mean *p*(FT) between .48 and .51 across parameter sets). The pattern was similar to the HN model for the EX data, with mean *p*(*χ* ^2^) = .21 and .36, and mean *p*(FT) = .56 and .51 for parameter sets 1 and 2, respectively. Results for the AL data stood out again, with a marked lack of fit for parameter set 1 (mean *p*(*χ* ^2^) = .02 and mean *p*(FT) = .74) but not for parameter set 2 (mean *p*(*χ* ^2^) = .44 and mean *p*(FT) = .50).

Spatial capture–recapture data sets simulated using the EX and AL detection functions, due to their longer right tails, occasionally resulted in very distant detections (> 9 du) from individual ACs. SCR models with HNP and HN detection models have difficulties to accommodate these distant detections. This is reflected in the posterior samples of detection probability for these detections, being of infinitesimal magnitude under the HNP model and hence minuscule Bayesian *p*‐value estimates *p*(*χ* ^2^).

## 4 DISCUSSION

Our study revealed that misspecifying the detection function in SCR studies can have potentially severe consequences for inference about animal space use. By contrast, and as previously reported, abundance/density estimates were nearly unaffected (Efford, 2004; Royle et al., 2014; Russell et al., 2012). Fortunately, misspecifications with the strongest impact on space use parameter estimates are also the ones most readily detectable using Bayesian *p*‐values. We also found that some detection functions are better able to accommodate a variety of space use patterns than others, with the half‐normal plateau and exponential function being the most and least flexible, respectively.

### 4.1 Consequences of misspecification

All misspecifications of the detection function led to pronounced bias in home range area estimates with the most common EX and HN models. Bias was especially severe when using a misspecified EX model (> 50% for all misspecifications, except for AL data with parameter set 1) and, to a lesser degree, a HN model. This finding is of concern, as the half‐normal is by far the most used detection function in SCR analyses (Royle et al., 2014). It is also noteworthy that a simple expansion of the half‐normal, the half‐normal plateau detection function, proved to be the most accommodating, due to the extra parameter (plateau width) which provides substantial additional flexibility.

On the contrary, and despite the wide range of space use patterns tested (Figure 2), we found little effect of misspecifications on the precision and accuracy of population size estimates. This corroborates findings from other studies that tested the consequences of misspecifying the detection function (Efford, 2004; Efford et al., 2009; Russell et al., 2012). Recently, Efford (2019) showed with simulations that SCR estimates of population size are also robust to some violations of the assumption of circularity of home ranges. In another study, Sutherland et al. (2015) had shown that accurate estimation of population size and home range geometry is possible using an ecological distance SCR model (instead of Euclidean distance) by explicitly modeling the species–landscape interactions when space use is not symmetrical.

In empirical applications, a misspecified detection function may also have indirect consequences for SCR‐based inferences if it leads to a smaller than required habitat buffer around the detector area. In populations that are geographically open beyond the study area, the size of the buffer is usually chosen to ensure virtually zero probability of detecting individuals with ACs outside the buffer. If the fitted detection function, also used for determining what this distance shall be (e.g., 3.5–4 times *σ* of the HN function, Royle et al., 2014) does not match the true process generating detections (e.g., EX function, which has a longer tail), population size estimates may be biased.

### 4.2 Goodness‐of‐fit

We found that misspecifications of the detection function can be challenging to detect using Bayesian *p*‐values. Among the four metrics used, only the FT and Pearson's *chi‐squared* were able to reveal some misspecifications. We found a positive correlation between the RB and *p*(*χ* ^2^), meaning that a model with a Bayesian *p*‐value > .5 is likely to overestimate home range area, while a model with a Bayesian *p*‐value < .5 is likely to underestimate it (Appendix S2: Figure S6). For example, under parameter set 1, HN models fitted to HNP data led to a mean *p*(*χ* ^2^) of .78 (with 2.5% and 97.5% quantiles as 0.67, 0.88 respectively) and to overestimated home range size estimates (21% average relative bias with 0% coverage prob.). Conversely, HN models fitted to AL data underestimated home range size (−14% average relative bias with 26% coverage prob.) and had a mean *p*(*χ* ^2^) of .04 (with 2.5% and 97.5% quantiles at 0 and 0.3; Figure 6).

Goodness‐of‐fit tests are aimed at identifying models with a poor fit. Using the recommended guidelines, that is, Bayesian *p*‐values falling outside the interval (.1, .9) indicate a lack of fit (mentioned in Section 2.7 and also in Royle et al., 2014), the Bayesian *p*‐values tested here would have failed to reveal most misspecifications, especially those based on the Freeman–Tukey metric. Indeed, our simulations showed that even *p*‐values between .20 and .80 do not guarantee that the detection function is correctly specified and that home range size estimates are free from bias. For instance, we obtained *p*(*χ* ^2^) of .78 and 21% relative bias in 95% home range size estimate for a HN model fitted to HNP data under parameter set 1, which illustrates the lack of power of the Bayesian *p*‐values tested here.

Nonetheless, the most problematic misspecifications could still be identified. For instance, in cases leading to the highest relative bias and lowest coverage probabilities (e.g., when fitting an EX model to data generated under another space use model), Bayesian *p*‐values were furthest from .5 and thus effective in identifying the lack of fit (Appendix S2: Figures S6 and S7).

Further, Bayesian *p*‐values performed substantially worse for parameter set 2 than for parameter set 1 as none of the misspecifications were identified even though home range area estimates showed moderate to severe bias. For example, under parameter set 2, mean relative bias of 95% home range size estimate was higher than 100% for a EX model fitted to AL or BI data, even though Bayesian *p*‐value in both cases was within the recommended interval (.1, .9). The lower number of detections in parameter set 2 is a likely explanation for this lack of power of Bayesian *p*‐value.

Practitioners should choose the Bayesian *p*‐value discrepancy metric with caution. As we have shown, different metrics perform differently. The FT metric with variance stabilizing property was overly optimistic in assessing GOF and showed high concentration around .5, even in cases of severe model misspecification. The pooled version of the discrepancy metrics (e.g., FT‐I and FT‐D) showed extremely high variance (Figure 6), and although they might be useful to identify other sources of lack of fit (Royle et al., 2014), they did not reveal misspecifications of the detection function in our study. Appropriate GOF metrics should be chosen based on their ability to detect certain misspecifications, instead of selecting and reporting those metrics that indicate a good fit (Head et al., 2015).

### 4.3 Recommendations

Based on our simulation study, we can make recommendations for SCR users regarding the choice of a detection function. First, density and population size estimates are largely immune to misspecifications of the detection function. However, if the goal is to estimate space use (e.g., home range size), SCR users need to be more cautious. If the SCR detection function does not match the space use pattern of the species under study, SCR‐based home range sizes are likely to be biased. This finding may partially explain the discrepancy between estimates of home range size obtained from SCR and collared individuals in previous studies (Bischof, Milleret, et al., 2020; Harmsen et al., 2020).

Bayesian *p*‐values near 0 or 1 are indicative a lack of fit and possible violation in underlying model assumptions. Furthermore, Bayesian *p*‐values can help reveal the direction of bias in home range size resulting from a misspecification, as we found a positive correlation between the relative bias in home range size and the Bayesian *p*‐values used in our study. However, *p*‐values close to the nominal value (.5, Stern & Cressie, 2000) do not necessarily indicate good fit. As a consequence, relying solely on Bayesian *p*‐values to diagnose model misspecification is too optimistic and we urge the development of additional diagnostic tools.

Testing SCR models’ goodness‐of‐fit is a key step toward reporting valid statistical inference and should be a precursor to model selection. Although it can often be impractical to fit alternative models, it is imperative to validate model adequacy using goodness‐of‐fit tests. When feasible, we recommend fitting multiple SCR models with different detection functions, computing associated Bayesian *p*‐values and also comparing their home range size estimates. Although relative bias and coverage probability cannot be assessed when models are fitted to empirical data, Bayesian *p*‐values can potentially reveal which of the fitted models are inadequate.

Our results suggest that the highest risk of flawed inferences is associated with the exponential detection function. If feasible, we recommend using a flexible detection function which can accommodate a variety of home range shapes. In our study, we have found the half‐normal plateau (HNP) detection function to be flexible enough to fit most home range shapes tested here. However, fitting the HNP detection function comes at the cost of estimating an additional parameter *w* and has proved to be more challenging to fit than the simpler half‐normal or exponential functions. Finally, alternative sources of information, such as telemetry data, may allow independent estimation of individual space use pattern in the study population (Royle, Chandler, Sun, et al., 2013) and inform the choice of detection function.

## 5 CONCLUSIONS

Although initially developed with the intent to provide spatially referenced estimates of population size (Efford, 2004), SCR is increasingly being used to estimate other ecological parameters that are sought after in wildlife management and conservation (Chandler et al., 2018; Ergon & Gardner, 2014). SCR holds particular promise for addressing spatial ecological questions at the population level with direct relevance for the monitoring and management of wide ranging populations (Bischof et al., 2017; Morin et al., 2017). Whereas SCR‐based population size and density estimates have proven to be remarkably robust to misspecifications of the detection function, repercussions can be severe when inferences about space use, for example, home range size, are sought. GOF testing should be an integral part of data analysis, especially when results have the potential to inform policy, and we urge for developing custom model checking tools to diagnose additional misspecifications of SCR models.

## CONFLICT OF INTEREST

It is hereby declared that the authors do not have any conflict of interest.

## AUTHOR CONTRIBUTIONS

**Soumen Dey:** Conceptualization (lead); Formal analysis (lead); Methodology (lead); Visualization (lead); Writing – original draft (lead); Writing – review & editing (lead). **Richard Bischof:** Conceptualization (equal); Formal analysis (supporting); Methodology (supporting); Validation (supporting); Writing – original draft (supporting); Writing – review & editing (supporting). **Pierre P. A. Dupont:** Conceptualization (supporting); Formal analysis (supporting); Methodology (supporting); Visualization (supporting); Writing – original draft (supporting); Writing – review & editing (supporting). **Cyril Milleret:** Conceptualization (supporting); Formal analysis (supporting); Methodology (supporting); Visualization (supporting); Writing – original draft (supporting); Writing – review & editing (supporting).

## Supporting information

Appendix S1‐S2

Data S1

Data S2

## Data Availability

No empirical data were used in this research. R codes for generating simulated data and SCR analysis are provided in the online supplementary material and also can be found in Zenodo (DOI: https://doi.org/10.5281/zenodo.5236502).
