# Supplementary material for: Does the punishment fit the crime? Consequences and diagnosis of misspecified detection functions in Bayesian spatial capture–recapture modeling
Source: Ecol Evol. 2022 Feb 15;12(2):e8600. doi: 10.1002/ece3.8600 (PMC8847120; doi:10.1002/ece3.8600)
Supplement: Supplementary file 1 — Appendix S1‐S2 [file ECE3-12-e8600-s003.pdf]

Appendix

Does the punishment fit the crime? Consequences and diagnosis of misspecified detection functions in Bayesian spatial capture-recapture modelling

Soumen Dey *[1], Richard Bischof[1], Pierre P. A. Dupont[1], and Cyril Milleret[1]

[1]Faculty of Environmental Sciences and Natural Resource Management, Norwegian University of Life Sciences, NO-1432 Ås, Norway

*E-mail: *soumenstat89@gmail.com* (✉)

Appendix S1 Tables of simulated SCR data summary, posterior estimates and Bayesian p-values

Table S1: Numbers (mean, median, 2.5% and 97.5% quantiles) of detected individuals and detections in simulated SCR data sets calculated over 50 repetitions for each pairing of simulated and fitted detection functions and for two sets of parameters.

Detection function (simulated)	Detection function (fitted)	Parameter Set	No. of detected individuals				No. of detections			
			Mean	Median	2.5% Quantile	97.5% Quantile	Mean	Median	2.5% Quantile	97.5% Quantile
HN	HN	1	123	123	111	138	405	400	337	473
EX	HN	1	136	134	122	148	401	406	352	455
HNP	HN	1	133	132	122	148	599	598	530	692
AL	HN	1	128	128	112	141	478	484	409	542
DN	HN	1	133	132	120	148	586	581	531	674
BI	HN	1	132	132	120	147	490	490	421	557
HN	HNP	1	123	124	113	131	408	405	362	468
EX	HNP	1	137	138	123	149	401	399	338	462
HNP	HNP	1	132	132	124	142	598	594	526	673
AL	HNP	1	127	128	114	139	475	474	412	536
DN	HNP	1	133	133	120	146	579	575	510	662
BI	HNP	1	133	132	120	148	489	498	415	544
HN	EX	1	123	124	108	137	407	407	339	473
EX	EX	1	137	138	124	146	406	412	344	463
HNP	EX	1	133	132	123	146	606	606	521	666
AL	EX	1	127	127	115	141	474	473	407	541
DN	EX	1	134	132	126	145	589	589	517	653
BI	EX	1	132	132	122	144	487	481	447	559
HN	HN	2	122	122	110	136	262	260	232	300
EX	HN	2	117	116	103	128	231	230	205	263
HNP	HN	2	127	127	113	139	301	300	255	331
AL	HN	2	127	127	115	140	429	426	363	488
DN	HN	2	126	126	113	138	290	284	258	339
BI	HN	2	121	122	104	135	292	292	246	331
HN	HNP	2	123	125	110	134	264	261	223	310
EX	HNP	2	117	118	103	131	233	234	204	270
HNP	HNP	2	127	127	115	138	306	302	269	369
AL	HNP	2	128	128	116	139	443	444	381	496
DN	HNP	2	125	125	116	137	293	295	258	339
BI	HNP	2	121	120	111	136	290	293	253	330
HN	EX	2	123	121	113	139	267	270	228	307
EX	EX	2	118	118	105	136	240	240	204	275
HNP	EX	2	126	125	115	137	305	304	265	342
AL	EX	2	126	126	114	138	431	428	368	477
DN	EX	2	126	124	115	137	295	292	259	330
BI	EX	2	122	123	106	135	300	296	253	354

Table S2: Relative bias (mean, median, 2.5% and 97.5% quantiles), coefficient of variation (mean, median, 2.5% and 97.5% quantiles) and coverage probability of the 95% credible interval for population size (N) calculated over 50 repetitions for each pairing of simulated and fitted detection functions and for two sets of parameters.

Detection function (simulated)	Detection function (fitted)	Parameter Set	Relative bias				Coefficient of variation				Coverage probability
			Mean	Median	2.5% Quantile	97.5% Quantile	Mean	Median	2.5% Quantile	97.5% Quantile	
HN	HN	1	0.020	0.020	−0.090	0.140	0.060	0.060	0.050	0.060	0.920
EX	HN	1	−0.010	0	−0.120	0.090	0.050	0.050	0.050	0.060	0.940
HNP	HN	1	0	−0.010	−0.080	0.110	0.050	0.050	0.050	0.050	0.940
AL	HN	1	0	0	−0.110	0.110	0.050	0.050	0.050	0.060	0.920
DN	HN	1	0	0	−0.090	0.110	0.050	0.050	0.050	0.050	0.940
BI	HN	1	0	−0.010	−0.090	0.120	0.050	0.050	0.050	0.060	0.920
HN	HNP	1	0.020	0.010	−0.070	0.090	0.060	0.060	0.060	0.060	1
EX	HNP	1	0	0.010	−0.110	0.100	0.050	0.050	0.050	0.060	0.920
HNP	HNP	1	0	0	−0.070	0.080	0.050	0.050	0.050	0.050	1
AL	HNP	1	−0.010	0	−0.110	0.090	0.050	0.050	0.050	0.060	0.960
DN	HNP	1	0.010	0.010	−0.090	0.120	0.050	0.050	0.050	0.050	0.960
BI	HNP	1	0.010	0	−0.080	0.120	0.050	0.050	0.050	0.060	0.940
HN	EX	1	0	0	−0.110	0.120	0.060	0.060	0.050	0.060	0.940
EX	EX	1	0.020	0.010	−0.060	0.090	0.050	0.050	0.050	0.060	0.980
HNP	EX	1	−0.020	−0.030	−0.090	0.080	0.050	0.050	0.050	0.050	0.980
AL	EX	1	−0.010	−0.010	−0.090	0.100	0.060	0.060	0.050	0.060	0.960
DN	EX	1	−0.010	−0.020	−0.070	0.060	0.050	0.050	0.050	0.050	1
BI	EX	1	−0.020	−0.010	−0.100	0.080	0.050	0.050	0.050	0.050	0.980
HN	HN	2	0.010	0.020	−0.150	0.120	0.070	0.070	0.060	0.080	0.920
EX	HN	2	−0.010	−0.020	−0.160	0.150	0.080	0.080	0.070	0.090	0.920
HNP	HN	2	0.020	0.030	−0.110	0.140	0.060	0.060	0.060	0.070	0.940
AL	HN	2	0	0	−0.080	0.090	0.060	0.060	0.050	0.060	0.960
DN	HN	2	0.030	0.030	−0.090	0.120	0.060	0.060	0.060	0.070	0.960
BI	HN	2	0.020	0.020	−0.150	0.160	0.070	0.060	0.060	0.070	0.900
HN	HNP	2	0	0	−0.130	0.140	0.070	0.070	0.060	0.080	0.960
EX	HNP	2	0	−0.010	−0.110	0.160	0.080	0.080	0.070	0.090	0.940
HNP	HNP	2	0.010	0.010	−0.100	0.100	0.060	0.060	0.060	0.070	1
AL	HNP	2	0.010	0.010	−0.100	0.090	0.050	0.050	0.050	0.060	0.980
DN	HNP	2	0	−0.010	−0.080	0.140	0.060	0.060	0.060	0.070	0.960
BI	HNP	2	0.030	0.010	−0.070	0.160	0.060	0.060	0.060	0.070	0.920
HN	EX	2	0.010	0	−0.090	0.150	0.070	0.070	0.060	0.080	0.960
EX	EX	2	0.020	0.010	−0.090	0.160	0.080	0.080	0.070	0.090	0.940
HNP	EX	2	0.010	0	−0.090	0.110	0.060	0.060	0.060	0.070	0.960
AL	EX	2	−0.020	−0.020	−0.120	0.090	0.060	0.060	0.050	0.060	0.940
DN	EX	2	0.020	0.020	−0.090	0.120	0.060	0.060	0.060	0.070	1
BI	EX	2	0.020	0.030	−0.120	0.140	0.060	0.060	0.060	0.070	0.940

Table S3: Relative bias (mean, median, 2.5% and 97.5% quantiles), coefficient of variation (mean, median, 2.5% and 97.5% quantiles), and coverage probability of the 95% credible interval for the kernel home range area (95% quantile) calculated over 50 repetitions for each pairing of simulated and fitted detection functions and for two sets of parameters.

Detection function (simulated)	Detection function (fitted)	Parameter Set	Relative bias				Coefficient of variation				Coverage probability
			Mean	Median	2.5% Quantile	97.5% Quantile	Mean	Median	2.5% Quantile	97.5% Quantile	
HN	HN	1	0.010	0.010	−0.110	0.140	0.060	0.060	0.060	0.070	0.940
EX	HN	1	−0.250	−0.250	−0.340	−0.080	0.070	0.070	0.060	0.080	0.060
HNP	HN	1	0.210	0.210	0.140	0.310	0.050	0.050	0.040	0.050	0
AL	HN	1	−0.140	−0.150	−0.280	0.050	0.060	0.060	0.050	0.060	0.260
DN	HN	1	0.230	0.230	0.130	0.340	0.050	0.050	0.040	0.050	0
BI	HN	1	0.260	0.260	0.150	0.380	0.060	0.060	0.050	0.070	0
HN	HNP	1	−0.040	−0.030	−0.150	0.050	0.070	0.070	0.060	0.080	0.900
EX	HNP	1	−0.260	−0.270	−0.390	−0.090	0.070	0.070	0.070	0.080	0.080
HNP	HNP	1	0.010	0.010	−0.090	0.140	0.060	0.060	0.050	0.060	0.960
AL	HNP	1	−0.150	−0.150	−0.270	−0.050	0.060	0.060	0.050	0.060	0.260
DN	HNP	1	−0.020	−0.020	−0.130	0.110	0.060	0.060	0.050	0.070	0.880
BI	HNP	1	0	0	−0.110	0.160	0.070	0.070	0.060	0.080	0.960
HN	EX	1	0.500	0.490	0.360	0.700	0.080	0.080	0.070	0.090	0
EX	EX	1	0.010	0	−0.130	0.170	0.090	0.090	0.080	0.100	0.960
HNP	EX	1	0.980	0.960	0.820	1.170	0.060	0.060	0.050	0.060	0
AL	EX	1	0.170	0.160	0.050	0.310	0.070	0.070	0.060	0.080	0.420
DN	EX	1	1.010	1	0.840	1.220	0.060	0.060	0.060	0.070	0
BI	EX	1	1	1.020	0.800	1.150	0.070	0.070	0.070	0.080	0
HN	HN	2	0	−0.020	−0.160	0.210	0.110	0.110	0.090	0.110	0.960
EX	HN	2	−0.220	−0.230	−0.410	0.020	0.120	0.120	0.110	0.140	0.380
HNP	HN	2	0.240	0.240	0.100	0.410	0.090	0.090	0.080	0.100	0.320
AL	HN	2	0.310	0.320	0.160	0.490	0.060	0.060	0.060	0.070	0
DN	HN	2	0.260	0.290	0.050	0.470	0.090	0.090	0.080	0.100	0.280
BI	HN	2	0.710	0.730	0.480	0.890	0.090	0.090	0.080	0.100	0
HN	HNP	2	−0.070	−0.070	−0.290	0.120	0.120	0.120	0.100	0.160	0.920
EX	HNP	2	−0.310	−0.310	−0.470	−0.140	0.120	0.120	0.110	0.150	0.100
HNP	HNP	2	0.040	0.060	−0.190	0.180	0.120	0.110	0.100	0.150	1
AL	HNP	2	0.070	0.060	−0.070	0.220	0.070	0.070	0.060	0.080	0.780
DN	HNP	2	0	0	−0.220	0.270	0.120	0.120	0.090	0.150	0.920
BI	HNP	2	0.010	0	−0.090	0.150	0.070	0.070	0.050	0.100	0.940
HN	EX	2	0.500	0.510	0.210	0.890	0.130	0.120	0.110	0.140	0.120
EX	EX	2	0.030	0.020	−0.220	0.260	0.140	0.130	0.120	0.160	0.960
HNP	EX	2	0.940	0.950	0.640	1.280	0.110	0.110	0.100	0.130	0
AL	EX	2	1.060	1.070	0.870	1.260	0.080	0.080	0.070	0.090	0
DN	EX	2	0.930	0.930	0.660	1.170	0.120	0.120	0.100	0.130	0
BI	EX	2	1.700	1.680	1.330	1.990	0.110	0.110	0.100	0.130	0

Table S4: Pearson's χ^2 and Freeman-Tukey (FT) Bayesian p-values (mean, median, 2.5% and 97.5% quantiles) calculated over 50 repetitions for each pairing of simulated and fitted detection functions and for two sets of parameters.

Detection function (simulated)	Detection function (fitted)	Parameter Set	Pearson's χ^2 Mean	Pearson's χ^2 Median	Pearson's χ^2 2.5% Quantile	Pearson's χ^2 97.5% Quantile	Freeman-Tukey Mean	Freeman-Tukey Median	Freeman-Tukey 2.5% Quantile	Freeman-Tukey 97.5% Quantile
HN	HN	1	0.500	0.520	0.280	0.630	0.480	0.480	0.450	0.550
EX	HN	1	0.180	0.170	0.010	0.410	0.560	0.560	0.530	0.590
HNP	HN	1	0.780	0.780	0.670	0.880	0.350	0.350	0.310	0.400
AL	HN	1	0.040	0.010	0	0.310	0.710	0.710	0.520	0.890
DN	HN	1	0.790	0.800	0.710	0.860	0.350	0.350	0.310	0.390
BI	HN	1	0.720	0.730	0.570	0.840	0.400	0.400	0.340	0.450
HN	HNP	1	0.440	0.450	0.140	0.610	0.500	0.500	0.470	0.540
EX	HNP	1	0.210	0.200	0.010	0.390	0.560	0.560	0.520	0.600
HNP	HNP	1	0.510	0.510	0.330	0.620	0.480	0.480	0.450	0.510
AL	HNP	1	0.020	0.010	0	0.150	0.740	0.740	0.600	0.880
DN	HNP	1	0.510	0.520	0.330	0.610	0.480	0.480	0.450	0.520
BI	HNP	1	0.520	0.530	0.430	0.590	0.490	0.490	0.470	0.510
HN	EX	1	0.840	0.860	0.760	0.930	0.320	0.320	0.270	0.400
EX	EX	1	0.490	0.500	0.220	0.730	0.500	0.500	0.430	0.540
HNP	EX	1	0.960	0.970	0.860	0.990	0.210	0.210	0.150	0.290
AL	EX	1	0.320	0.160	0	0.840	0.430	0.440	0.320	0.550
DN	EX	1	0.950	0.960	0.870	0.990	0.190	0.200	0.150	0.230
BI	EX	1	0.920	0.930	0.860	0.970	0.230	0.230	0.200	0.270
HN	HN	2	0.480	0.500	0.270	0.580	0.510	0.510	0.490	0.530
EX	HN	2	0.350	0.360	0.120	0.510	0.520	0.520	0.500	0.530
HNP	HN	2	0.590	0.590	0.470	0.690	0.490	0.490	0.460	0.520
AL	HN	2	0.670	0.690	0.400	0.800	0.410	0.410	0.370	0.450
DN	HN	2	0.590	0.590	0.500	0.680	0.490	0.490	0.470	0.510
BI	HN	2	0.690	0.690	0.620	0.740	0.460	0.460	0.430	0.480
HN	HNP	2	0.470	0.480	0.350	0.530	0.510	0.510	0.490	0.530
EX	HNP	2	0.360	0.370	0.130	0.490	0.510	0.510	0.490	0.540
HNP	HNP	2	0.510	0.510	0.430	0.560	0.500	0.500	0.480	0.520
AL	HNP	2	0.440	0.470	0.190	0.560	0.500	0.500	0.480	0.520
DN	HNP	2	0.510	0.520	0.430	0.540	0.500	0.500	0.490	0.520
BI	HNP	2	0.520	0.520	0.480	0.540	0.510	0.510	0.480	0.520
HN	EX	2	0.700	0.700	0.560	0.800	0.480	0.480	0.450	0.500
EX	EX	2	0.520	0.520	0.330	0.680	0.500	0.500	0.480	0.520
HNP	EX	2	0.790	0.800	0.690	0.860	0.430	0.430	0.400	0.460
AL	EX	2	0.890	0.890	0.820	0.940	0.260	0.260	0.220	0.290
DN	EX	2	0.790	0.790	0.670	0.860	0.440	0.440	0.400	0.460
BI	EX	2	0.820	0.820	0.750	0.890	0.400	0.400	0.350	0.430

Appendix S2 Additional figures

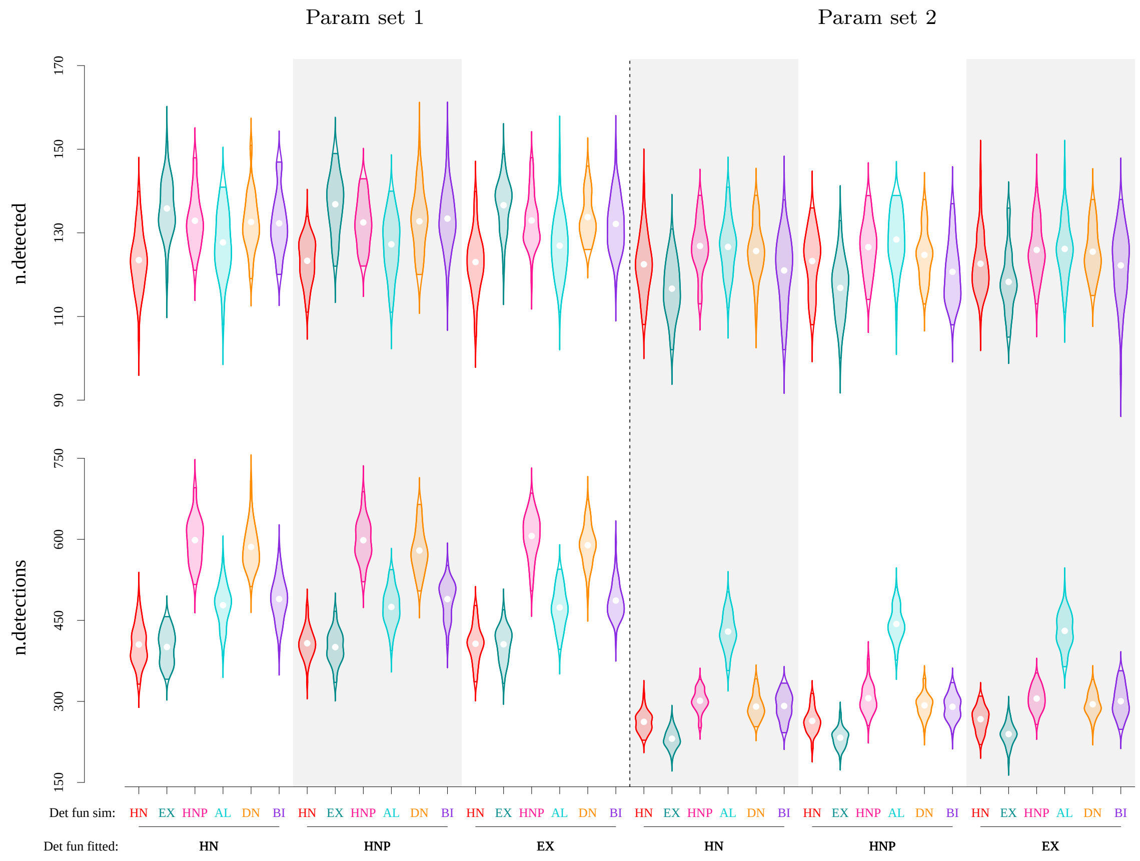

Figure S1: Comparison of number of detected individuals (in panel 1) and total number of detections across all the individuals (i.e., $\sum_{i=1}^{M}\sum_{j=1}^{J} y_{ij}$, in panel 2) from the simulated SCR data sets of each of the 36 scenarios. Violin of a particular colour (e.g., red) in the graph correspond to a particular detection function (e.g., 'HN' for red) which was used to simulate the SCR data sets (indexed in first row of labels along x-axis). The second row of labels represents the detection functions that was used to fit the SCR models.

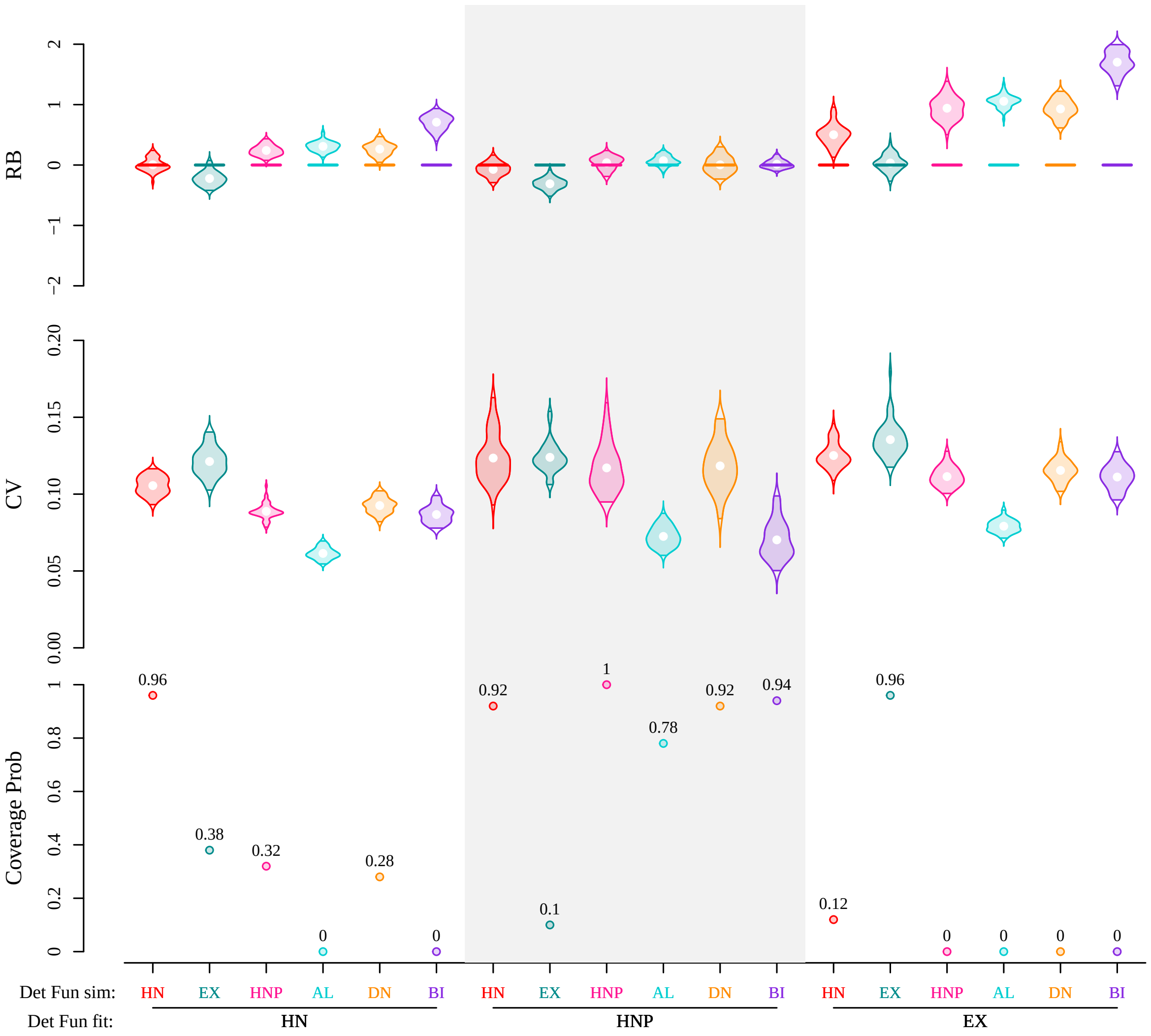

Figure S2: Relative bias (RB), coefficient of variation (CV), and 95% coverage probability of the home range size estimator for each pairing of simulated (Det Fun sim) and fitted (Det Fun fit) detection functions with parameter set 2. Home range area was estimated as the 95% kernel of the estimated detection function. Detection functions include the half-normal (HN), exponential (EX), half-normal plateau (HNP), asymmetric logistic (AL), donut (DN) and bimodal (BI). RB/CV violins represent the distribution over 50 simulations.

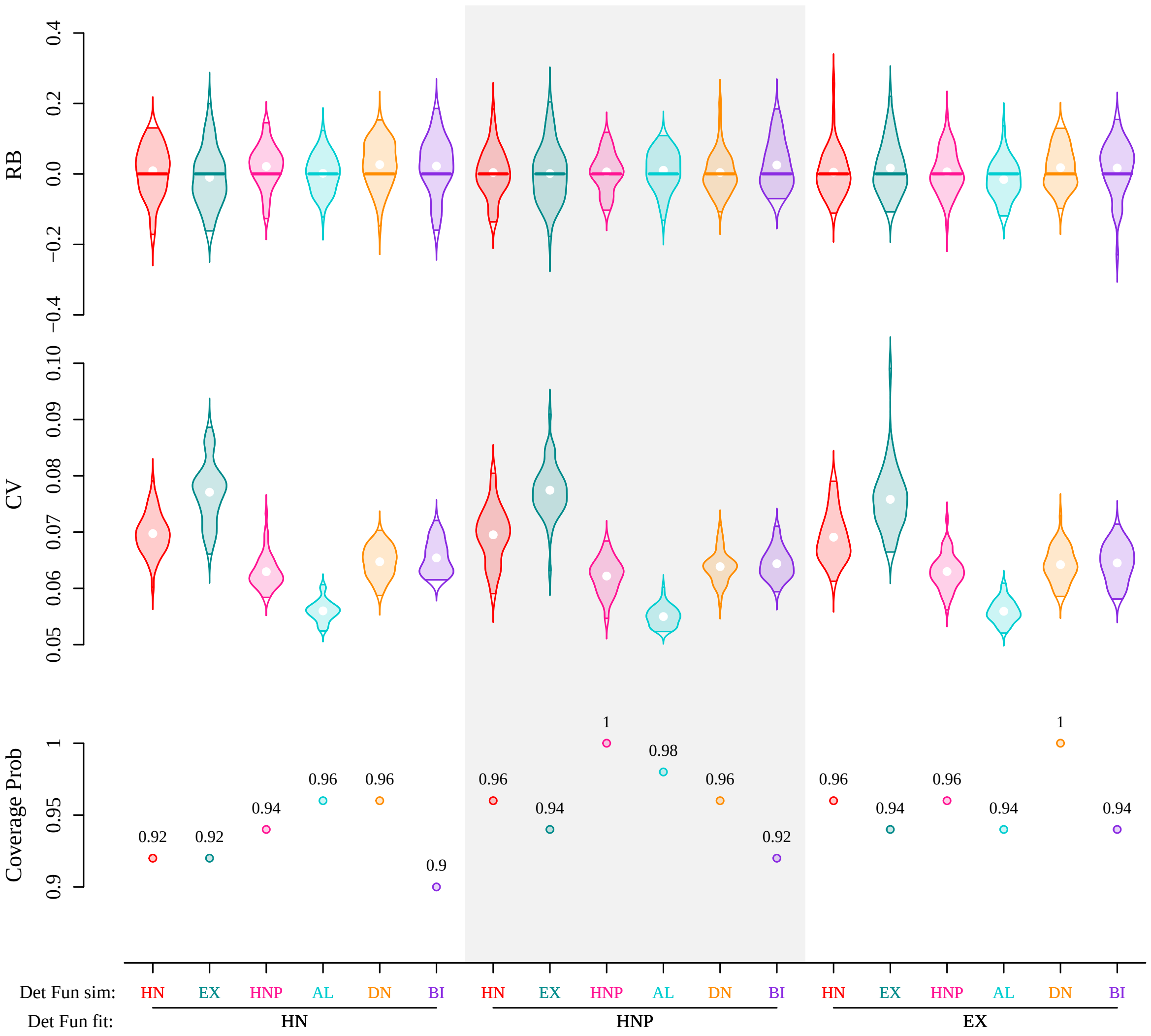

Figure S3: Relative bias (RB), coefficient of variation (CV), and 95% coverage probability of the population size estimator N for each pairing of simulated (Det Fun sim) and fitted (Det Fun fit) detection functions with parameter set 2. Detection functions include the half-normal (HN), exponential (EX), half-normal plateau (HNP), asymmetric logistic (AL), donut (DN) and bimodal (BI). RB/CV violins represent the distribution over 50 simulations

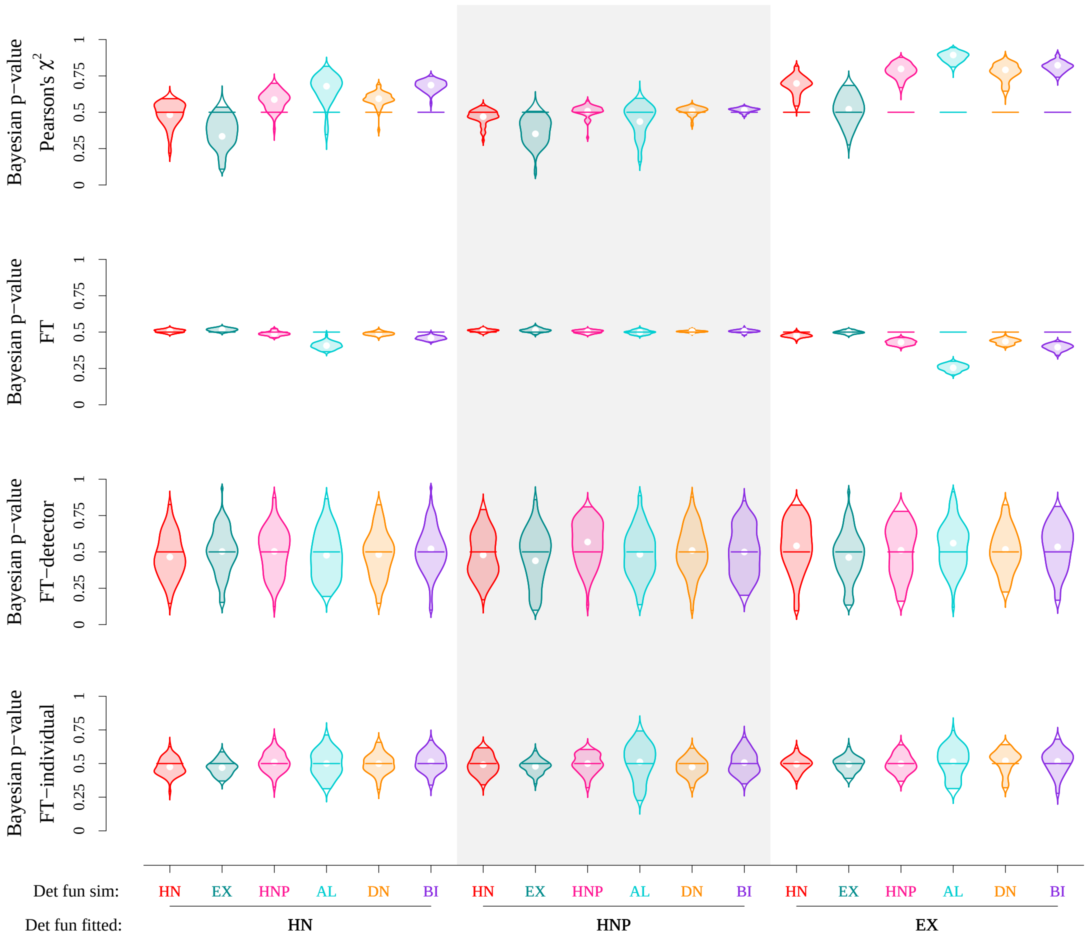

Figure S4: Bayesian p-values based on different metrics (Freeman-Tukey (FT), Pearson's χ^2, FT metric based on individual level count (FT-I) and FT metric based on detection level count (FT-D)) for each pairing of simulated (Det Fun sim) and fitted (Det Fun fit) detection functions with parameter set 2. Each Violin represents the distribution of Bayesian p-values over 50 simulations.

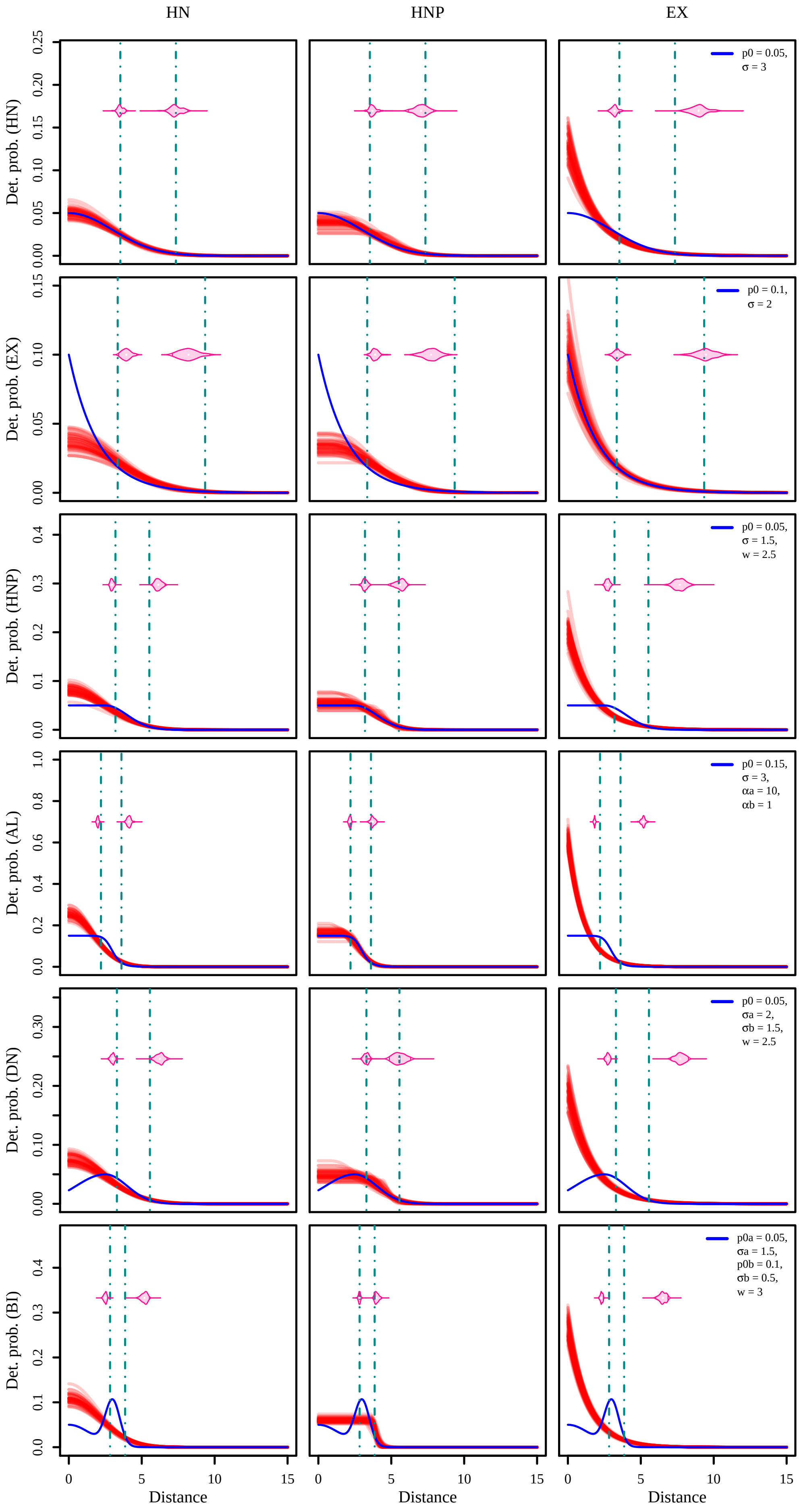

Figure S5: Comparison of simulated ('blue' line) and estimated detection functions ('red' lines) for parameter set 2. 'pink' violins represent the distribution of the 50% and 95% home range radius estimates over 50 repetitions and'deep cyan' vertical lines represent the simulated 50% and 95% home range radius. Rows correspond to detection functions used for simulation and columns correspond to detection functions used to fit the SCR model: HN, HNP and EX.

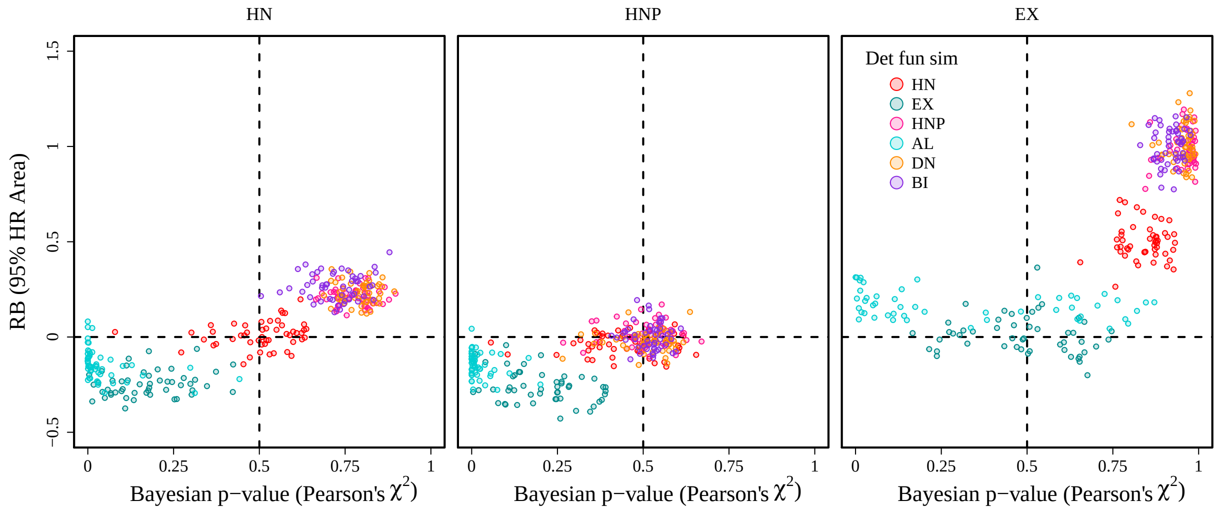

(a) Parameter set 1

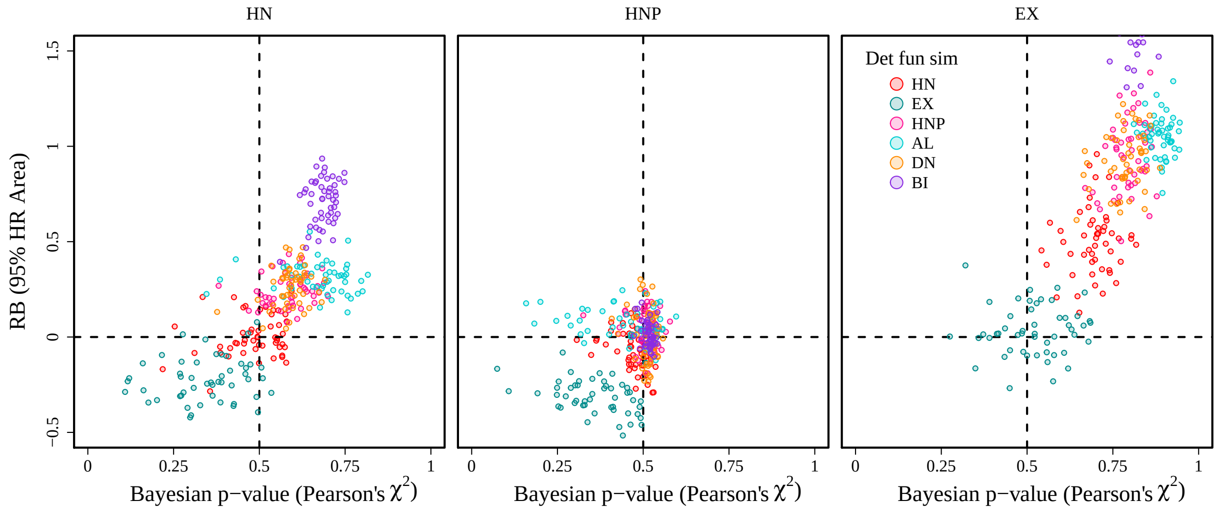

(b) Parameter set 2

Figure S6: Scatter plots of Pearson's χ^2 Bayesian p-values against the relative bias of home range size estimates (95% kernel of the estimated detection function) for parameter sets 1 (in first row) and 2 (in second row). Colours correspond to the different detection functions used for simulation: the half-normal (HN), exponential (EX), half-normal plateau (HNP), asymmetric logistic (AL), donut (DN) and bimodal (BI). Columns correspond to the detection functions that were used to fit the SCR model. Dotted horizontal line indicate the reference value 0 for the relative bias and dotted vertical line indicate the reference value 0.5 for the Bayesian p-value.

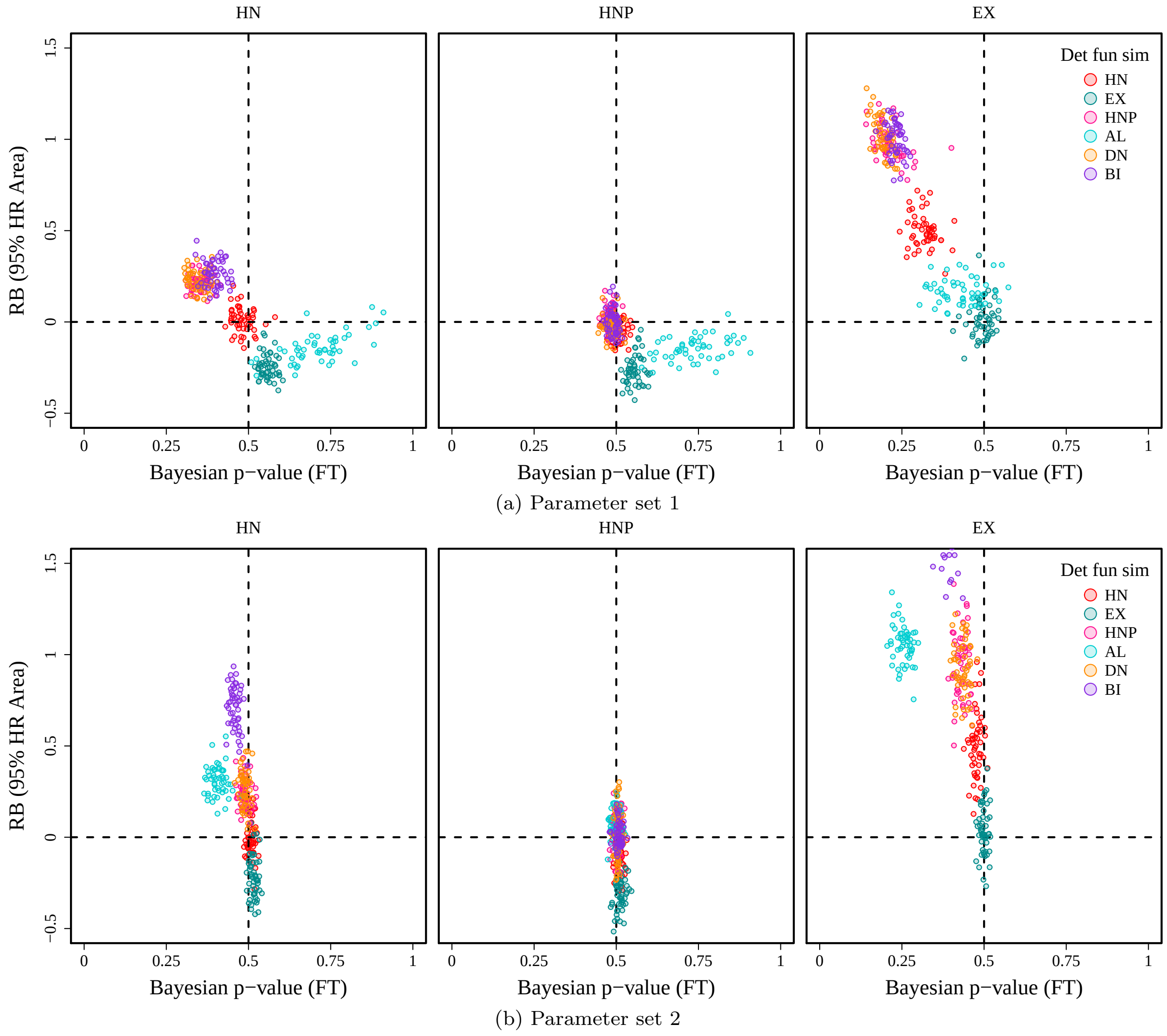

Figure S7: Scatter plots of Freeman-Tukey Bayesian p-values against the relative bias of home range size estimates (95% kernel of the estimated detection function) for parameter sets 1 (in first row) and 2 (in second row). Colours correspond to the different detection functions used for simulation: the half-normal (HN), exponential (EX), half-normal plateau (HNP), asymmetric logistic (AL), donut (DN) and bimodal (BI). Columns correspond to the detection functions that were used to fit the SCR model. Dotted horizontal line indicate the reference value 0 for the relative bias and dotted vertical line indicate the reference value 0.5 for the Bayesian p-value.

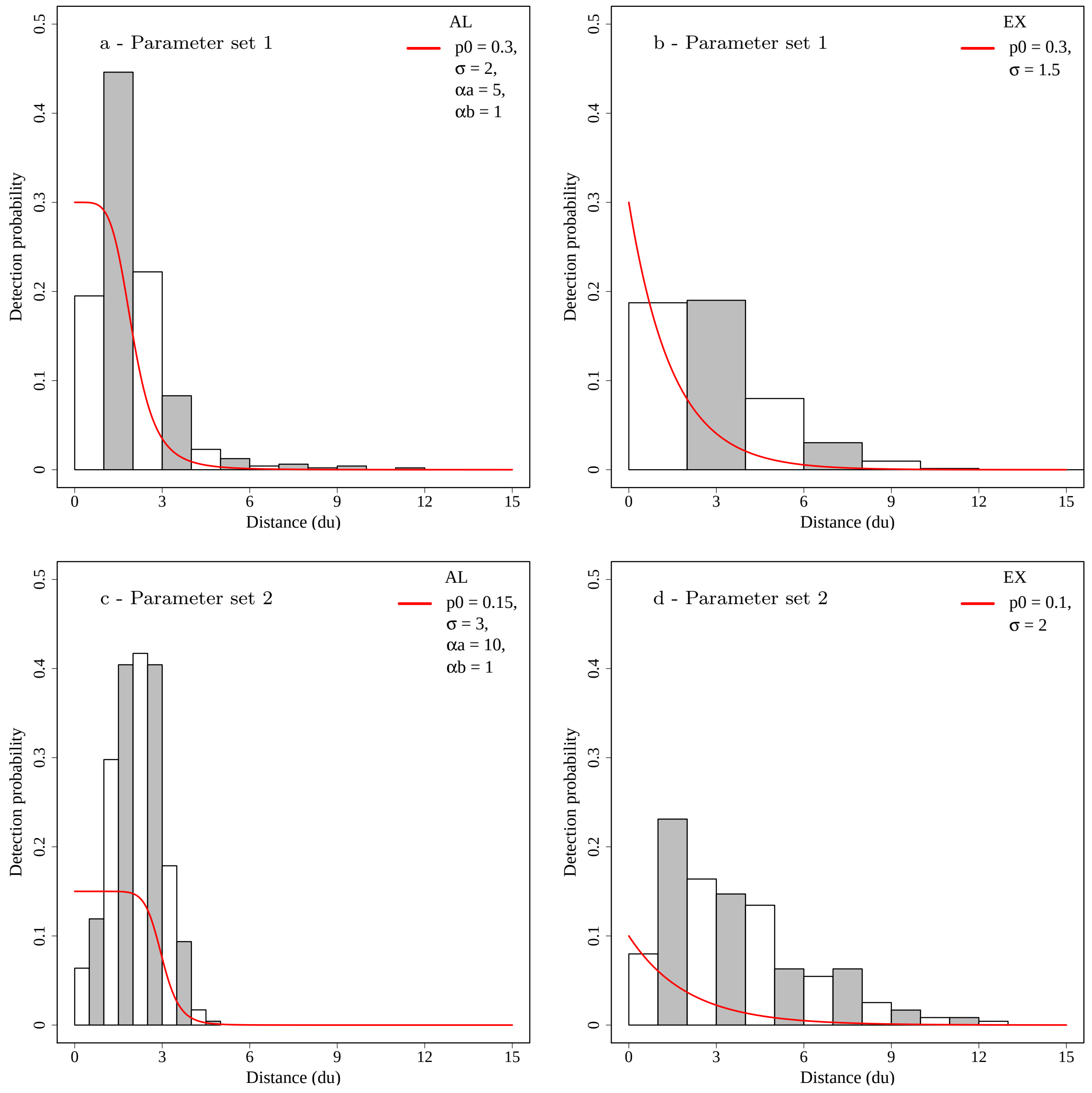

Figure S8: Histograms of distances between the ACs and their corresponding detection locations of the detected individuals in example SCR data sets simulated under asymmetric logistic (Plots a and c) and exponential (Plots b and d) detection functions. The red line indicates the curve for the corresponding detection function under parameter sets 1 and 2. Plots a and d reveal the presence of detections in the histogram at distances > 9 du. HN or HNP model are unlikely to be able to accommodate these distant detections. As a result, the posterior samples of detection probability corresponding to the pairing of individual and detector are of infinitesimal magnitude under HN or HNP models and hence Bayesian p-value estimates $p(\chi^2)$ approach 0.
